# Synthesis of ribavirin 2’-Me-*C*-nucleoside analogues

**DOI:** 10.3762/bjoc.13.74

**Published:** 2017-04-21

**Authors:** Fanny Cosson, Aline Faroux, Jean-Pierre Baltaze, Jonathan Farjon, Régis Guillot, Jacques Uziel, Nadège Lubin-Germain

**Affiliations:** 1 Laboratoire de Chimie Biologique, University of Cergy-Pontoise, 5 mail Gay-Lussac, Cergy-Pontoise, France; 2 ICMMO, UMR CNRS 8182, University of Paris Sud, 15 rue Georges Clémenceau, Orsay, France; 3 Laboratoire CEISAM, UMR 6230, University of Nantes, 2 rue de la Houssinière, Nantes, France

**Keywords:** alkynylation, antiviral, cancer, C-nucleosides, ribavirin

## Abstract

An efficient synthetic pathway leading to two carbonated analogues of ribavirin is described. The key-steps in the synthesis of these ribosyltriazoles bearing a quaternary carbon atom in the 2’-position are an indium-mediated alkynylation and a 1,3-dipolar cyclization.

## Introduction

**Figure 1:**
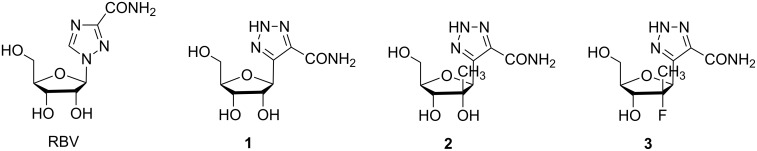
Targeted compounds.

The triazole nucleoside ribavirin (RBV, Figure 1) is used for the treatment of a number of viral infections and may be promising as an anticancer drug [1–3]. The antiviral activity of ribavirin is ascribed to a combination of different mechanisms [4]. Although RBV causes some side effects [5–7] essentially due to its accumulation in red blood cells, it is indispensable in the treatment against hepatitis C virus (HCV). The current standard-of-care for hepatitis C involves taking a combination [8] of an antipolymerase compound (sofosbuvir [9–10]) and an antiprotease compound (simeprevir [11–12]), both associated to ribavirin. If the presence of interferon is not required for the therapy, ribavirin is mandatory in the combination, due to its particular role.

Recently, we developed an alkynyl glycosylation protocol allowing us to obtain *C*-nucleoside derivatives and we turned our attention to ribavirin *C*-nucleoside analogues. Moreover, recently De Clerq [13] outlined the potential of *C*-nucleosides in the arsenal of antivirals due to their stability in biological fluids and their bioavailability.

**Figure 2:**
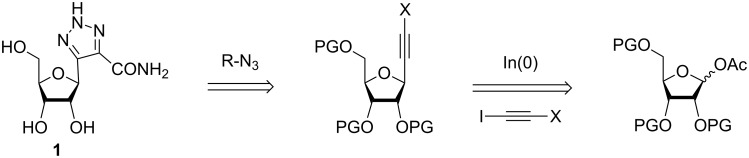
Retrosynthesis of compound **1**.

Some years ago, we described an approach leading to the *C*-ribosylated analogue **1** of ribavirin (Figure 2) with the key-steps of the synthesis being an indium-mediated alkynylation of a ribose derivative followed by the Huisgen cycloaddition reaction onto the *C*-alkynyl riboside intermediately obtained [14].

Herein we describe the synthesis of two new carbonated analogues **2** and **3** of RBV modified at the 2’-position (Figure 1). In fact, a quaternization in 2’-position of various nucleosides led to a higher efficacy against HCV as in the case of 2’-*C*-methylcytidine and 2’-*C*-methyladenosine [15]. The structure–activity studies of 2’-*C*-methylnucleosides showed that the methyl substituent must be in 2’-position and on the β face for an optimal efficacy that drops when the methyl is on the α face or in the 3’-position or if a bulkier ethyl group is used [16]. On the other hand, currently 2’-deoxy-2’-*C*-methyl-2’-*C-*fluoronucleosides are developed because a fluoro substituent in the 2’-position increases the antiviral activity and specificity due to a higher tolerance of viral polymerases with respect to incorporation of such compounds [17]. In clinical studies (phase I and II), the fluorinated compound mericitabine in combination with PEG-IFN and RBV was better tolerated and more effective in genotype 1 or 4 patients compared to the standard combination of Peg-IFN and RBV [18–19].

Further, the therapy of untreated patients with HCV genotype 1, 2, or 3 infections with a combination of sofosbuvir (Gilead) and ribavirin for 12 weeks is considered as the most effective treatment at the moment [20].

## Results and Discussion

**Scheme 1:**
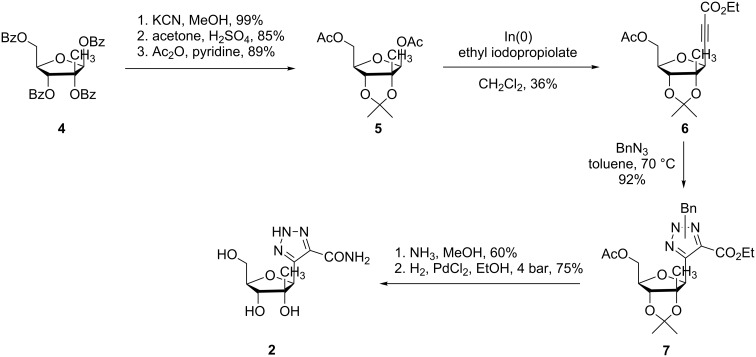
Synthesis of 5-(2’-*C*-methyl-β-D-ribofuranosyl)-1,2,3-triazole-4-carboxamide (**2**).

2’-*C*-Methylnucleoside **2** was synthesized according to a seven step pathway starting from the commercially available 2-*C*-methyl-1,2,3,5-tetra-*O*-benzoyl-β-D-ribofuranose (**4**) as shown in Scheme 1. Debenzoylation of **4** followed by selective protection led to derivative **5**, which was submitted to the indium-mediated alkynylation reaction affording the alkynyl riboside **6** with the same β-anomeric selectivity as for the non-methylated derivative [21]. Then, the 1,3-dipolar cycloaddition reaction of **6** with benzyl azide in toluene at 70 °C led to a mixture of regioisomeric triazoles **7** in a 42:58 ratio. The removal of all protecting groups was achieved by treatment of compounds **7** with ammonia followed by catalytic hydrogenolysis. The latter reaction simultaneously cleaves the benzyl and isopropylidene groups affording compound **2** as a single isomer [22].

In the case of 5-(2’-deoxy-2’-methyl-2’-fluoro-β-D-ribosyl)-1,2,3-triazole-4-carboxamide (**3**) the synthesis was more delicate as it is necessary to differentiate the 2’ position.

**Scheme 2:**
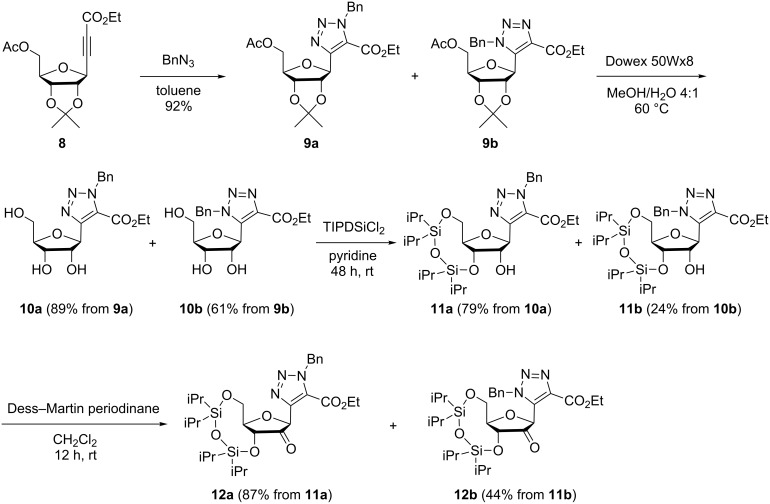
Synthesis of the 2’-keto derivatives **12a/12b**.

After the indium-mediated alkynylation, the obtained alkynyl riboside **8** was submitted to a Huisgen cycloaddition reaction with benzyl azide, under the same conditions as in the previous case, affording the mixture of regioisomeric triazoles **9a** and **9b** in a 37:63 ratio [14] (Scheme 2).

The selective protection of the 3’ and 5’-positions requires the full deprotection of the ribose. This was performed by the treatment with Dowex 50Wx8 (H^+^) in methanol. However, carrying out this step with the mixture of **9a**/**9b** the reaction led to only a moderate yield (52%) and with formation of a partially deprotected compound (only acetal deprotected), stemming from **9b**, demonstrating the different hydrolysis rate of each regioisomer. An HPLC analysis of the disappearance of **9a** and **9b** and the formation of **10a** and **10b** showed a ratio of 2:1 in favor of regioisomer **10a**. The hydrolysis was more efficient when performed with the isolated **9a** or **9b** isomers. In this case, the fully deprotected compounds **10a** and **10b** were obtained in 89% (after 36 h) and 61% (after 2 weeks) yields, respectively.

This rate difference was also observed in the subsequent protection of the 3’,5’ positions with 1,3-dichloro-1,1,3,3-tetraisopropyldisiloxane (TIPDSCl_2_). While this reaction proceeded with a very poor yield in the case of the mixture **10a**/**10b** (~10% yield), compound **11a** was obtained in 79% yield from pure **10a** (24% yield for **11b** starting from **10b**). Thereafter, the oxidation of **11a** to the corresponding ketone with Dess–Martin periodinane afforded **12a** in 87% yield whereas the reaction of the less reactive isomer **11b** led to **12b** in 44% yield.

**Figure 3:**
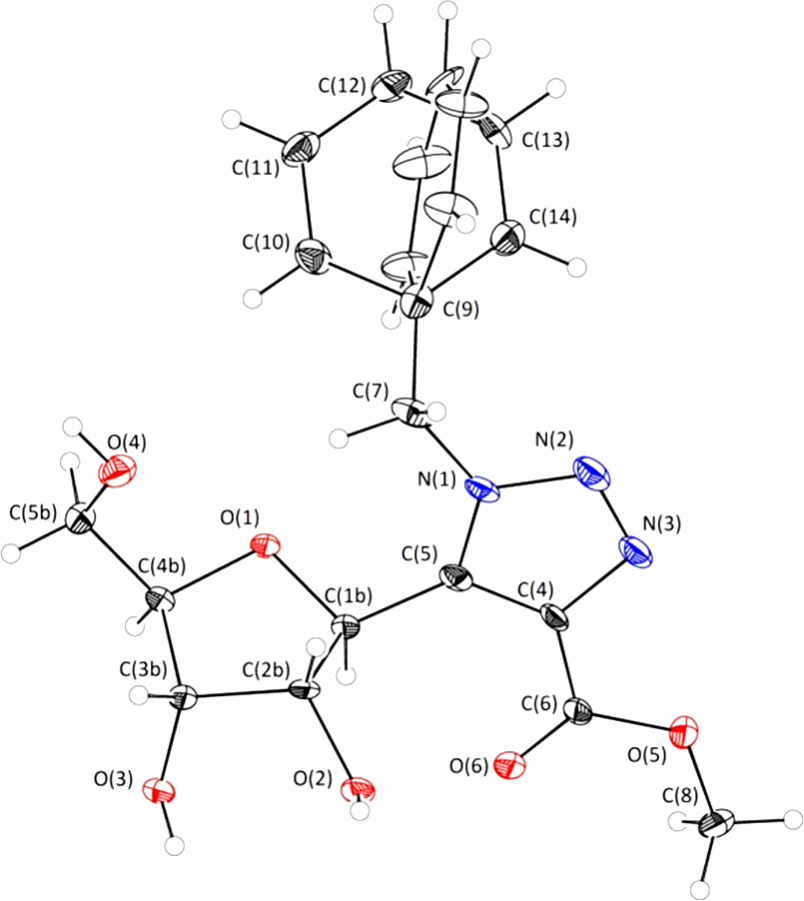
X-ray spectrum of compound **10b**.

With the aim to get some explanations for the different reactivities observed for the two isomers **10a** and **10b**, we investigated the structure of the less reactive compound **10b**. As depicted in Figure 3, compound **10b** displayed an S-type conformation and an *anti* arrangement of the atoms O(1’)-C(1’)-C(1)-N(2) according to the dihedral angle of 0° lower than 90° (43°). Moreover, the benzyl group appeared to be present in two different positions covering the furan ring.

For the attempted methylation of the 2’-position different conditions were tested (Table 1). As described for other nucleosides [23], the use of MeLi led to compound **13b** obtained by an attack from the α-face even if this proceeded with a very low yield (7%). The use of MeMgBr gave an almost 1:1 mixture by α- and β-attack; this second one can be explained by a magnesium complexation with the base. More interestingly, the methylation proceeded stereoselectively leading to **13b** in 87% yield when trimethylaluminium was used [24].

**Table 1:** Methylation of ketone **12**.

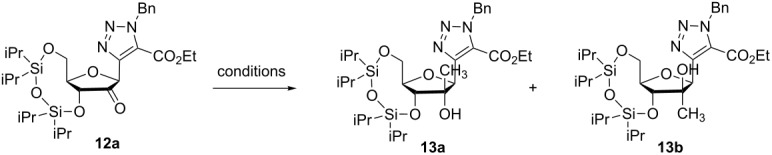			

	Conditions	**13a**	**13b**

MeMgBr	CH_2_Cl_2_, 30 min, rt	19	15
MeLi	Et_2_O, 30 min, rt	traces	7
AlMe_3_	CH_2_Cl_2_, 1 h, rt	–	87

**Figure 4:**
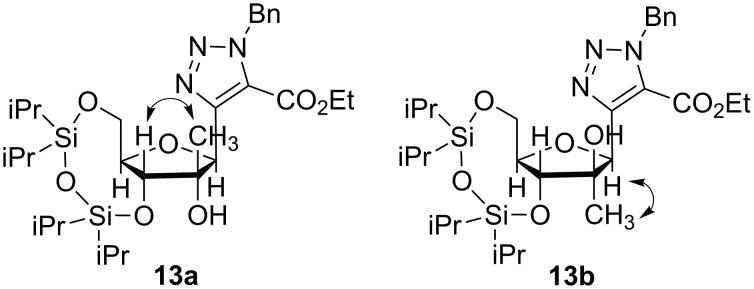
Structural study of isomeric compounds **13**.

The stereochemical outcome of this reaction was determined by selective 1D NOESY experiments (Figure 4). First, the hydrogen H3’ in **13a** and **13b** was selectively excited. The nOes observed for compound **13a** are in the following order of decreasing intensity: CH_3_ > H5’_a_ > H5’_b_-H4’ > H1’ confirming that CH_3_ and H3’ are spatially close. In the case of **13b** the nOe intensities decrease in the order: H5’_b_-H4’ > CH_3_ > H1’. The selective excitation of H1’ in **13a** led to nOes with decreasing intensities in the order: H5’_b_-H4’ > CH_3_ > H3’, whereas for **13b** the order was CH_3_ > H5’_b_-H4’. This second series of nOes confirms that CH_3_ is closer to H1’ in **13b** than in **13a**.

**Scheme 3:**
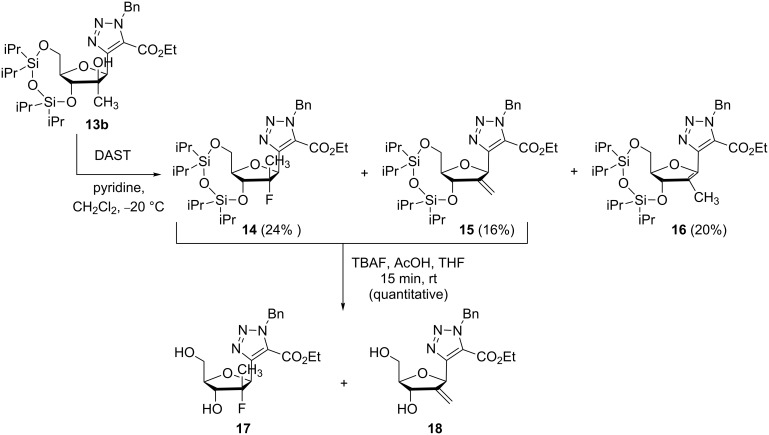
Fluorination of ethyl 1-benzyl-4-(2’-C-methyl-3’,5’-O-(tetraisopropyldisiloxane-1,3-diyl)-β-D-ribofuranosyl)-1,2,3-triazole-5-carboxylate (**13b**).

The fluorination of **13b** with DAST was performed at −20 °C and afforded the desired fluorinated derivative **14** (24%) along with two elimination products, the exocyclic olefin **15** (16%) and the corresponding endocyclic one **16** (20%). As it was impossible to separate **15** from **14** at this stage, the mixture **14**/**15** was deprotected with tetrabutylammonium fluoride leading to the mixture of diols **17** and **18** in quantitative yield which were easily separated (Scheme 3).

**Scheme 4:**
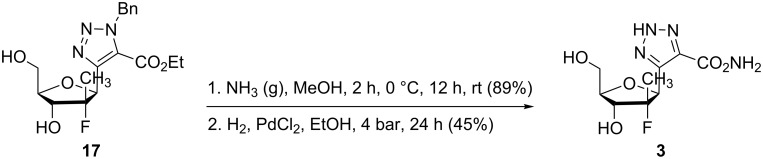
Synthesis of 5-(2’-deoxy-2’-fluoro-2’-methyl-β-D-ribofuranosyl)-1,2,3-triazole-4-carboxamide (**3**).

Finally, the aminolysis of compound **17** followed by catalytic hydrogenolysis in the presence of palladium chloride led to the desired compound **3** [22] (Scheme 4).

## Conclusion

The indium-mediated alkynylation of a ribose derivative followed by a Huisgen cyclization allowed the access to 2’-quaternized carbonated analogues of ribavirin. While the synthesis of compound **2** starting from 2-methylated ribose derivative **4** was quite easy to perform, the preparation of the fluorinated analogue **3** required a more complicated pathway. This included the selective protection of the 3’,5’ positions, the stereoselective methylation and the fluorination of the 2’ position. The synthesized compounds are currently investigated for their antiviral activities.

## Experimental

Experimental procedures for compounds **2**, **5–7**, and **10–18** are covered by the Ph.D. thesis of Fanny Cosson [22].

**5-(2’-*C*-Methyl-β-D-ribofuranosyl)-1,2,3-triazole-4-carboxamide (2)** [22]: Through the solution of compound **7** (mixture of **7a** and **7b**, 0.49 g, 1.1 mmol) in anhydrous methanol (14 mL) was bubbled ammonia gas for 2 h at 0 °C. Then the mixture was stirred for 12 h at rt and concentrated in vacuum. The residue was purified by flash chromatography (EtOAc/cyclohexane, 3:7 to 1:0) affording the mixture of the corresponding 1-benzyl-4-(2’,3’-*O*-isopropylidene-2’-*C*-methyl-β-D-ribofuranosyl)-1,2,3-triazole-5-carboxamide and 1-benzyl-5-(2’,3’-*O*-isopropylidene-2’-*C*-methyl-β-D-ribofuranosyl)-1,2,3-triazole-4-carboxamide (0.25 g, 60%) in a 27:73 ratio as an oil. IR (cm^−1^) ν_max_: 3336, 2360, 2342, 1635; ^1^H NMR (400 MHz, CDCl_3_) δ 7.32–7.25 (m, 5H, Ph), 6.05 (d, *J* = 14.6 Hz, 1H, CH_2_Ph), 5.59 (d, *J* = 14.6 Hz, 1H, CH_2_Ph), 5.37 (s, 1H, H1), 4.54 (d, *J* = 5.0 Hz, 1H, H3), 4.35–4.32 (m, 1H, H4), 3.82–3.75 (m, 2H, H5, H5’), 1.60 (s, 3H, CH_3_), 1.37 (s, 3H, C(CH_3_)_2_), 1.34 (s, 3H, C(CH_3_)_2_) and δ 7.32-7.25 (m, 5H, Ph), 6.12 (d, *J* = 14 Hz, 1H, CH_2_Ph), 5.80 (d, *J* = 14 Hz, 1H, CH_2_Ph), 5.33 (s, 1H, H1), 4.84–4.81 (m, 1H, H4), 4.45 (d, *J* = 2.7 Hz, 1H, H3), 3.91 (dd, *J* = 3.7 Hz, 11.9 Hz, 1H, H5), 3.82–3.75 (m, 1H, H5’), 1.64 (s, 3H, CH_3_), 1.24 (s, 3H, C(CH_3_)_2_), 1.23 (s, 3H, C(CH_3_)_2_); ^13^C NMR (100 MHz, CDCl_3_) δ 160.3 (CONH_2_), 149.7 (triazole), 129.6 (Cq Ph), 129–128 (Ph), 125.0 (triazole), 112.9 (C(CH_3_)_2_), 90.0 (C2), 87.1 (C3), 85.1 (C4), 84.1 (C1), 62.0 (C5), 53.8 (CH_2_Ph), 26.6 (C(CH_3_)_2_), 26.5 (C(CH_3_)_2_), 14.3 (CH_3_) and δ 159.1 (CONH_2_), 141.7 (triazole), 135.6 (Cq Ph), 135.1 (triazole), 129–128 (Ph), 114.5 (C(CH_3_)_2_), 90.7 (C2), 87.8 (C3), 84.8 (C4), 82.4 (C1), 62.8 (C5), 54.0 (CH_2_Ph), 26.0 (C(CH_3_)_2_), 25.3 (C(CH_3_)_2_); HRMS calcd for C_19_H_22_O_5_, 330.1467; found, 330.1478.

The mixture of carboxamides and palladium chloride (23 mg, 0.13 mmol) in ethanol (10 mL) was hydrogenated with H_2_ at 4 bar for 48 h. After filtration over Celite and concentration in vacuum, the crude was purified by flash chromatography (cyclohexane/EtOAc 2:8 to EtOAc/MeOH 1:1) to afford compound **2** as a white solid (0.12 g, 75%) that was lyophilized. [α]_D_^24^ −47 (*c* 1, MeOH); mp 185–190 °C; IR (cm^−1^) ν_max_: 3202, 1663, 1604, 1382, 1014; ^1^H NMR (400 MHz, MeOD) δ 5.48 (s, 1H, H1), 4.11–4.04 (m, 1H, H4), 3.99 (d, *J* = 8.3 Hz, 1H, H3), 3.83 (dd, *J* = 2.7 Hz, 12.4 Hz, 1H, H5), 3.64 (dd, *J* = 3.6 Hz, 11.9 Hz, 1H, H5’), 1.22 ppm (s, 3H, CH_3_); ^13^C NMR (100 MHz, MeOD) δ 164.1 (CONH_2_), 137.8 (triazole), 82.4 (C4), 78.5 (C1), 78.1 (C2), 76.2 (C3), 61.6 (C5), 19.9 (CH_3_); HRMS calcd for C_9_H_15_N_4_O_5_, 259.1042; found, 259.1055.

**5-(2’-Deoxy-2’-fluoro-2’-methyl-β-D-ribofuranosyl)-1,2,3-triazole-4-carboxamide (3)** [22]: Through the solution of compound **17** (110 mg, 0.29 mmol) in anhydrous methanol (4 mL) was bubbled ammonia for 2 h at 0 °C. After stirring at rt overnight, the solution was concentrated in vacuum affording 1-benzyl-4-(2’-deoxy-2’-fluoro-2’-methyl-β-D-ribofuranosyl)-1,2,3-triazole-5-carboxamide (90 mg, 89%) as a white powder. [α]_D_^25^ −18.6 (*c* 0.9, MeOH); mp 176 °C; IR (cm^−1^) ν_max_: 3397, 3304, 2944, 2883, 1668, 1598, 1458, 1218, 1126, 1048; ^1^H NMR (400 MHz, MeOD) δ 7.25–7.18 (m, 5H, Ph), 5.85 (d, *J* = 14.6 Hz, 1H, CH_2_Ph), 5.73 (d, *J* = 14.6 Hz, 1H, CH_2_Ph), 5.42 (d, *J* = 24.8 Hz, 1H, H1), 4.14 (dd, *J* = 8.7 Hz, 18.8 Hz, 1H, H3), 3.97–3.93 (m, 1H, H4), 3.90 (dd, *J* = 2.8 Hz, 12.4 Hz, 1H, H5), 3.72 (dd, *J* = 5.0 Hz, 12.4 Hz, 1H, H5’), 1.18 (d, *J* = 22.5 Hz, 3H, CH_3_); ^13^C NMR (100 MHz, MeOD) δ 160.8 (CONH_2_), 143.7 (d, *J* = 11.4 Hz, triazole), 135.3 (Cq Ph), 130.4 (triazole), 128.6, 128.2, 127.7 (Ph), 100.8 (d, *J* = 190 Hz, C2), 82.2 (C4), 79.7 (d, *J* = 39 Hz, C1), 73.9 (d, *J* = 23 Hz, C3), 61.3 (C5), 53.0 (-CH_2_Ph), 17.2 (d, *J* = 26 Hz, CH_3_); ^19^F NMR (376.2 MHz, MeOD) −158.2 (m); HRMS calcd for C_16_H_20_FN_4_O_4_, 351.1469; found, 351.1468.

Debenzylation proceeded in ethanol (5 mL) with palladium chloride (9.1 mg, 0.05 mmol) under 4 bar pressure of hydrogen for 24 h. After filtration over Celite and concentration in vacuum, the crude was purified by flash chromatography (pure EtOAc to EtOAc/MeOH 1:1) to afford **3** as a white solid (0.03 g, 45%) that was lyophilized. [α]_D_^25^ +18.5 (*c* 0.7, MeOH); mp 52 °C; IR (cm^−1^) ν_max_ 3357, 2361, 2341, 1635, 1455, 1072, 1021; ^1^H NMR (400 MHz, MeOD) δ 5.80 (d, *J* = 22 Hz, 1H, H1), 4.06–3.98 (m, 3H, H3, H4, H5), 3.84 (dd, *J* = 2.8 Hz, 11.9 Hz, 1H, H5’), 1.13 (d, *J* = 24.8 Hz, 3H, CH_3_); ^13^C NMR (100 MHz, MeOD) δ 163.8 (-CONH_2_), 137.9 (triazole), 101.2 (d, *J* = 174 Hz, C2), 81.5 (C4), 78.5 (d, *J* = 39 Hz, C1), 72.6 (d, *J* = 23 Hz, C3), 60.6 (C5), 16.3 (d, *J* = 26 Hz, CH_3_); ^19^F NMR (376.2 MHz, MeOD) −160.8 (m); HRMS calcd for C_9_H_14_FN_4_O_4_, 261.0999; found, 261.1006.

## Supporting Information

File 1 Experimental details, copies of ^1^H NMR, ^13^C NMR, HRMS and X-ray spectra.
