# Supplementary material for: Synthesis of ribavirin 2’-Me-C-nucleoside analogues
Source: Beilstein J Org Chem. 2017 Apr 21;13:755–61. doi: 10.3762/bjoc.13.74 (PMC5405694; doi:10.3762/bjoc.13.74)
Supplement: File 1 — Experimental details, copies of 1H NMR, 13C NMR, HRMS and X-ray spectra. [file Beilstein_J_Org_Chem-13-755-s001.pdf]

Supporting Information

for

Synthesis of ribavirin 2'-Me-*C*-nucleoside analogues

Fanny Cosson[1], Aline Faroux[1], Jean-Pierre Baltaze[2], Jonathan Farjon[3], Régis Guillot[2], Jacques Uziel*[1] and Nadège Lubin-Germain*[1]

Address: [1]Laboratoire de Chimie Biologique, University of Cergy-Pontoise, 5 mail Gay-Lussac, Cergy-Pontoise, France, [2]ICMMO, UMR CNRS 8182, University of Paris Sud, 15 rue Georges Clémenceau, Orsay, France and [3]Laboratoire CEISAM, UMR 6230, University of Nantes, 2 rue de la Houssinière, Nantes, France

Email: Nadège Lubin-Germain - nadege.lubin-germain@u-cergy.fr; Jacques Uziel - jacques.uziel@u-cergy.fr

*Corresponding author

Experimental details, copies of ^{1}H NMR, ^{13}C NMR, HRMS and X-ray spectra

Table of contents

Experimental part S2
Spectroscopic data for **5** S14
Spectroscopic data for **6** S15
Spectroscopic data for **7a/7b** S16
Spectroscopic data for **2** S17
Spectroscopic data for **10a** S18
Spectroscopic data for **10b** S19
Spectroscopic data for **11a** S20
Spectroscopic data for **11b** S21
Spectroscopic data for **12a** S22
Spectroscopic data for **12b** S23
Spectroscopic data for **13a** S24
Spectroscopic data for **13b** S27
Spectroscopic data for **14/15** S30
Spectroscopic data for **16** S32
Spectroscopic data for **17** S33
Spectroscopic data for **18** S34
Spectroscopic data for **3** S35
X-Ray analysis for **10b** S36
References S37

Experimental

General methods

All starting materials were obtained from commercial suppliers and used as received. The ^{1}H, ^{13}C and ^{19}F NMR spectra were recorded on a JEOL ECX 400 spectrometer and chemical shifts are reported with tetramethylsilane or C_6F_6 as an internal standard. IR spectra were recorded on a Bruker Tensor infrared spectrometer. Melting points were measured on a Büchi apparatus without correction. HRMS spectra were recorded on JEOL JMS-GC Mate II spectrometer. Optical rotation was measured with a JASCO P1010 polarimeter.

General statement

Experimental procedures for compounds **2**, **5–7**, and **10–18** are covered by the PhD thesis of Fanny Cosson [1].

1,5-*O*-Diacetyl-2,3-*O*-isopropylidene-2-*C*-methyl-D-ribofuranoside (5)

To 2-*C*-methyl-1,2,3,5-tetra-*O*-benzoyl-β-D-ribofuranose (**4**, 9.74 g, 16.75 mmol) in anhydrous MeOH (200 mL) was added KCN (30 mg, 0.45 mmol) and the mixture stirred at rt overnight. Concentration in vacuum and drying over P_2O_5 afforded 2-*C*-methyl-D-furanose in quantitative yield (2.82 g). The latter compound was dissolved in anhydrous acetone (200 mL) containing H_2SO_4 (200 μL) and the mixture stirred overnight at rt before being neutralised with sat. $NaHCO_3$ and concentrated in vacuum. The residue was dissolved in EtOAc (100 mL) and washed with water and sat. NaCl. The aqueous layer was extracted with EtOAc and the combined organic layers dried over $MgSO_4$, filtered and concentrated in vacuum affording 2,3-*O*-isopropylidene-2-*C*-methyl-D-furanose as a yellow oil (3 g, 85%). ^{1}H NMR (400 MHz,

$CDCl_3$): δ 5.22 (s, 1H, H1), 4.45-4.30 (m, 1H, H3), 4.30-4.10 (m, 1H, H4), 3.80-3.50 (m, 2H, H5, H5'), 1.43 (s, 3H, CH_3), 1.42 (s, 3H, CH_3), 1.34 (s, 3H, CH_3). ^{13}C NMR (100 MHz, $CDCl_3$): δ 112.5 (Cq), 104.0 (C1), 92.3 (C2), 87.4 (C3), 82.4 (C4), 63.3 (C5), 28.0 (CH_3), 27.7 (CH_3), 19.7 (CH_3). Acetylation proceeded by overnight stirring with acetic anhydride (70 mL) and pyridine (70 mL) followed by concentration under vacuum. Compound **5** was obtained as yellow oil (3.78 g, 89%). $[\alpha]_D^{26}$ –6.12 (c = 1.8, $CHCl_3$). IR ν_{max} (cm^{-1}) 2988, 2940, 2360, 1742,1456, 1215, 1009, 858. ^1H NMR (400 MHz, $CDCl_3$) δ 6.08 (s, 1H, H1), 4.31 (dt, *J* = 0.9 Hz, 7 Hz, 1H, H4), 4.27 (d, *J* = 0.9 Hz, 1H, H3), 4.09 (dd, *J* = 7 Hz, 11 Hz, 1H, H5), 4.04 (dd, *J* = 7 Hz, 11 Hz, 1H, H5'), 2.03 (s, 3H, Ac), 2.02 (s, 3H, Ac), 1.42 (s, 3H, $C(CH_3)_2$), 1.41 (s, 3H, CH_3),1.38 (s, 3H, $C(CH_3)_2$). ^{13}C NMR (100 MHz, $CDCl_3$) δ 170.3 Ac), 169.4 (Ac), 113.5 ($\underline{C}(CH_3)_2$), 102.9 (C1), 91.5 (C2), 87.5 (C3), 84.6 (C4), 64.0 (C5), 28.0 ($C(CH_3)_2$), 27.6 ($C(CH_3)_2$), 21.3 (Ac), 20.8 (Ac), 20.5 (CH_3). HRMS: calcd for $C_{13}H_{20}O_7$: 288.1209; Found: 288.1207.

Ethyl 3-(5-*O*-acetyl-2,3-*O*-isopropylidene-2-*C*-methyl-β-D-ribofuranosyl) propiolate (6)

To a sealed tube containing activated indium (0.95 g, 8.3 mmol) was added compound **5** (1 g, 3.47 mmol) and ethyl iodopropiolate (1.56 g, 6.94 mmol) in anhydrous DCM (18 mL) under stirring and the mixture was stirred for 36 h at 55 °C. After filtration over Celite and washing with EtOAc, the filtrate was concentrated in vacuum and the residue was purified by flash chromatography (EtOAc/cyclohexane 1:4 to 1:1) affording compound **6** (0.405 g, 36%) as a yellow oil. $[\alpha]_D^{26}$ –59 (c = 1.5, $CHCl_3$). IR ν_{max} (cm^{-1}) 2988, 2363, 1743, 1374, 1240, 1094. ^1H NMR (400 MHz, $CDCl_3$) δ 4.59 (s, 1H, H1), 4.38 (dt, *J* = 1.8 Hz, 4.8 Hz, 1H, H4), 4.32 (d, *J* = 1.8 Hz,

1H, H3), 4.22 (q, J = 7 Hz, 2H, Et), 4.20 (dd, J = 4.8 Hz, 12 Hz, 1H, H5), 4.15 (dd, J = 4.8 Hz, 12 Hz, 1H, H5'), 2.09 (s, 3H, Ac), 1.58 (s, 3H, -C(CH_3)$_2$), 1.56 (s, 3H, CH_3), 1.42 (s, 3H, -C(CH_3)$_2$), 1.29 (t, J = 7 Hz, 3H, Et). ^{13}C NMR (100 MHz, $CDCl_3$) δ 170.3 (Ac), 153.2 (-CO_2Et), 115.1 (-C(CH_3)$_2$), 90.6 (C2), 87.6 (C3), 82.4 (C4), 81.6 (C≡), 80.3 (C≡), 77.7 (C1), 63.7 (C5), 62.2 (Et), 27.4 (-C(CH_3)$_2$), 26.9 (-C(CH_3)$_2$), 23.5 (CH_3), 20.9 (Ac), 14.1 (Et). HRMS calcd for $C_{16}H_{23}O_7$: 327.1444; Found: 327.1455.

Ethyl 1-benzyl-4-(5'-*O*-acetyl-2',3'-*O*-isopropylidene-2'-*C*-methyl-β-D-ribofuranosyl)-1,2,3-triazole-5-carboxylate (7a) and ethyl 1-benzyl-5-(5'-*O*-acetyl-2',3'-*O*-isopropylidene-2'-*C*-methyl-β-D-ribofuranosyl)-1,2,3-triazole-4-carboxylate (7b)

Compound **6** (0.347 g, 1.06 mmol) and benzyl azide (0.566 mg, 4.26 mmol) in toluene (4 mL) were stirred at 70 °C for 24 h. After concentration in vacuum the crude was purified by flash chromatography (EtOAc/cyclohexane 1:4 to 1:1) affording the mixture of compounds **7a** and **7b** (0.38 g, 92%) in a 42:58 ratio as a yellow oil. IR ν_{max} (cm^{-1}) 2358, 2340, 1743, 1381, 1210, 1100. ^{1}H NMR (400 MHz, $CDCl_3$) **7a**: δ 7.29-7.21 (m, 5H, Ph), 5.84 (d, J = 1.8 Hz, 2H, CH_2Ph), 5.39 (s, 1H, H1), 4.53 (s, 1H, H3), 4.95-4.87 (m, 1H, H4), 4.43-4.12 (m, 4H, H5, H5', Et), 2.08 (s, 3H, Ac), 1.64-1.19 (m, 12H, C(CH_3)$_2$, Et, CH_3); **7b**: δ 7.29-7.21 (m, 5H, Ph), 6.01 (s, 1H, H1), 5.99 (d, J = 14 Hz, 1H, CH_2Ph), 5.60 (d, J = 14 Hz, 1H, CH_2Ph), 4.43-4.38 (m, 1H, H4), 4.35 (d, J = 1.8 Hz, 1H, H3), 4.43-4.12 (m, 4H, H5, H5', Et), 2.09 (s, 3H, Ac), 1.64-1.19 (m, 12H, C(CH_3)$_2$, Et, CH_3). ^{13}C NMR (100 MHz, $CDCl_3$) **7a**: δ 170.8 (Ac), 158.6 (CO_2Et), 149.0 (triazole), 135.2 (Ph), 129-127 (Ph), 125.2 (triazole), 114.6 (C(CH_3)$_2$), 90.6 (C2), 88.2 (C3), 81.9 (C1, C4), 61.9 (C5), 60.5 (Et), 54.0 (CH_2Ph), 27.2 (C(CH_3)$_2$), 26.1 (C(CH_3)$_2$), 25.2 (CH_3), 20.8 (Ac), 14.4 (Et); **7b**: δ 170.5 (Ac), 161.7

(CO_2Et), 136.5 (triazole), 136.0 (triazole), 135.2 (Ph), 129-127 (Ph), 113.3 ($C(CH_3)_2$), 91.9 (C2), 88.1 (C3), 81.9 (C4), 79.6 (C1), 64.1 (C5), 61.3 (Et), 54.7 (CH_2Ph), 27.5 ($C(CH_3)_2$), 26.7 ($C(CH_3)_2$), 22.8 (CH_3), 20.9 (Ac), 14.3 (Et). HRMS calcd for $C_{23}H_{29}N_3O_7Na$: 482.1903; Found: 482.1987.

Ethyl 1-benzyl-4-(β-D-ribofuranosyl)-1,2,3-triazole-5-carboxylate (10a)

The mixture of compound **9a** [2] (5.4 g, 12.1 mmol) in MeOH (430 mL) and water (110 mL) was stirred with Dowex 50Wx8 (40 g) at 60 °C for 36 h. After filtration and concentration in vacuum, the residue was purified by flash chromatography with EtOAc to afford **10a** as a yellow solid (3.9 g, 89%) as an oil. $[\alpha]_D^{25}$ –15 (c = 1.1, MeOH). M.p. = 96°C. IR ν_{max} (cm^{-1}) 3269, 2928, 2499, 1722, 1256, 1029. ^1H NMR (400 MHz, MeOD) δ 7.31-7.24 (m, 5H, Ph), 5.91 (s, 2H, CH_2Ph), 5.36 (d, *J* = 4.6 Hz, 1H, H1'), 4.36 (q, *J* = 6.8 Hz, 2H, $CO_2CH_2CH_3$), 4.31 (t, *J* = 5.0 Hz, 1H, H2'), 4.23 (t, *J* = 5.5 Hz, 1H, H3'), 3.99 (dd, *J* = 4.6 Hz, 8.7 Hz, 1H, H4'), 3.82 (dd, *J* = 3.2 Hz, 12.4 Hz, 1H, H5'a), 3.66 (dd, *J* = 4.6 Hz, 12.4 Hz, 1H, H5'b), 1.32 (t, *J* = 6.8 Hz, 3H, CH_3). ^{13}C NMR (100 MHz, MeOD) δ 158.3 (-CO_2Et), 149.5 (triazole), 135.9 (Ph), 128.5, 128.0, 127.4 (Ph), 125.7 (-triazole), 84.8 (C4'), 76.8 (C1'), 75.8 (C2'), 71.1 (C3'), 62.1 (C5'), 62.0 (CH_2CH_3), 53.6 (CH_2Ph), 13.0 (CH_3). HRMS calcd for $C_{17}H_{22}N_3O_6$: 364.1509; Found: 364.1510.

Ethyl 1-benzyl-5-(β-D-ribofuranosyl)-1,2,3-triazole-4-carboxylate (10b)

The mixture of compound **9b** [2] (0.5 g, 1.12 mmol) in MeOH (40 mL) and water (10 mL) was stirred with Dowex 50Wx8 (5 g) at 60 °C for 2 weeks. After filtration and concentration in vacuum, the residue was purified by flash chromatography with EtOAc to afford **10b** as a yellow solid (0.25 g, 61%). $[\alpha]_D^{25}$ +20 (c = 1.1, MeOH). IR

ν_{max} (cm^{-1}) 3357, 2360, 2342, 1653, 1457, 1210, 1048. ^{1}H NMR (400 MHz, MeOD) δ 7.35-7.26 (m, 3H, Ph), 7.23-7.19 (m, 2H, Ph), 5.89 (q, *J* = 15.6 Hz, 2H, CH_2Ph), 5.53 (d, *J* = 7.8 Hz, 1H, H1), 4.38 (q, *J* = 7.3 Hz, 2H, Et), 4.07-3.99 (m, 2H, H2 et H3), 3.90 (dd, *J* = 4.1 Hz, 6.9 Hz, 1H, H4), 3.77 (dd, *J* = 2.7 Hz, 11.9 Hz, 1H, H5), 3.71 (dd, *J* = 4.1 Hz, 11.9 Hz, 1H, H5'), 1.38 (t, *J* = 6.9 Hz, 3H, Et). ^{13}C NMR (100 MHz, $CDCl_3$) δ 161.1 (-CO_2Et), 138.8 (triazole), 137.2 (triazole), 135.7 (Cq Ph), 128.5, 127.9, 127.0 (Ph), 86.7 (C4), 75.0 (C2), 74.8 (C1), 70.7 (C3), 61.5 (C5), 61.2 (Et), 52.7 (-CH_2Ph), 13.2 (Et). HRMS calcd for $C_{17}H_{22}N_3O_6$: 364.1509; Found: 364.1513.

Ethyl 1-benzyl-4-(3',5'-*O*-(tetraisopropyldisiloxane-1,3-diyl)-β-D-ribofuranosyl)-1,2,3-triazole-5-carboxylate (11a)

To a mixture of compound **10a** (2.45 g, 6.75 mmol) and anhydrous pyridine (90 mL) was added under argon atmosphere 1,3-dichloro-1,1,3,3-tetraisopropyldisiloxane (3.16 mL, 10.1 mmol). After stirring at rt for 2 days and concentration in vacuum, the residue was purified by flash chromatography (EtOAc/cyclohexane 0.5:9.5 to 2:8) to afford **11a** as a white solid (3.22 g, 79%). $[\alpha]_D^{24}$ –45 (c = 2.5, MeOH). m.p. 78-82°C. IR ν_{max} (cm^{-1}) 2945, 2867, 1728, 1464, 1036, 885. ^{1}H NMR (400 MHz, MeOD) δ 7.26-7.19 (m, 5H, Ph), 5.80 (s, 2H, CH_2Ph), 5.33 (d, *J* = 2.8 Hz, 1H, H1), 4.70 (t, *J* = 5.9 Hz, 1H, H3), 4.49-4.46 (m, 1H, H2), 4.28 (q, *J* = 7.3 Hz, 2H, Et), 3.98-3.87 (m, 3H, H4, H5, H5'), 1.28 (t, *J* = 7.3 Hz, 3H, Et), 1.05-0.95 (m, 28H, -$CH(CH_3)_2$). ^{13}C NMR (100 MHz, $CDCl_3$) δ 158.5 (-CO_2Et), 148.9 (triazole), 135.0 (Cq Ph), 128.8, 128.4, 128.1 (Ph), 125.4 (triazole), 82.6 (C4), 77.3 (C1), 75.3 (C2), 73.9 (C3), 63.5 (C5), 62.2 (Et), 53.9 (-CH_2Ph), 17.6-17.1 (-$CH(CH_3)_2$), 14.1-12.7 (-$CH(CH_3)_2$, Et). HRMS calcd for $C_{29}H_{47}N_3O_7Si_2$: 605.2953; Found: 605.2941.

Ethyl 1-benzyl-5-(3',5'-*O*-(tetraisopropyldisiloxane-1,3-diyl)-β-D-ribofuranosyl)-1,2,3-triazole-4-carboxylate (11b)

The same procedure as described for **11a**, starting from **10b** (0.45 g, 1.23 mmol), pyridine (12 mL) and 1,3-dichloro-1,1,3,3-tetraisopropyldisiloxane (0.6 mL, 1.84 mmol) led to **11b** as a yellow oil (0.18 g, 24%). $[\alpha]_D^{25}$ +0.3 (c = 2, $CHCl_3$). IR ν_{max} (cm^{-1}) 3447, 2944, 2867, 2360, 2342, 1724, 1559, 1247, 1041. ^1H NMR (400 MHz, $CDCl_3$) 7.30-7.26 (m, 3H, Ph), 7.15-7.12 (m, 2H, Ph), 5.93 (d, *J* = 15.6 Hz, 1H, CH_2Ph), 5.67 (d, *J* = 15.1 Hz, 1H, CH_2Ph), 5.49 (d, *J* = 4.1 Hz, 1H, H1), 4.42 (q, *J* = 6.8 Hz, 2H, Et), 4.18 (t, *J* = 6.9 Hz, 1H, H3), 4.04-4.02 (m, 2H, H5, H5'), 3.99-3.95 (m, 1H, H2), 3.93-3.89 (m, 1H, H4), 1.41 (t, *J* = 6.8 Hz, 3H, Et), 1.09-0.96 (m, 28H, -$CH(CH_3)_2$). ^{13}C NMR (100 MHz, $CDCl_3$) δ 161.5 (-CO_2Et), 138.9 (triazole), 137.0 (triazole), 135.7 (Cq Ph), 128.9, 128.3, 127.0 (Ph), 82.9 (C4), 78.9 (C1), 75.6 (C2), 71.3 (C3), 61.6 (C5), 61.5 (Et), 53.7 (-CH_2Ph), 17.6-17.0 (-$CH(CH_3)_2$), 14.4-12.6 (-$CH(CH_3)_2$, Et). HRMS calcd for $C_{29}H_{48}N_3O_7Si_2$: 606.3031; Found: 606.3036.

Ethyl 1-benzyl-4-(3',5'-*O*-(tetraisopropyldisiloxane-1,3-diyl)-β-D-erythropentofuran-2-ulosyl)-1,2,3-triazole-5-carboxylate (12a)

To a solution of compound **11a** (2.95 g, 4.88 mmol) in anhydrous DCM (90 mL) was added under argon Dess–Martin periodinane (3.06 g, 9.77 mmol). After stirring overnight at rt, the solution was filtered through a 1:1 mixture of silica gel/sodium sulfate and washed with ethyl ether. The filtrate was washed with sat. sodium thiosulfate, sat. sodium hydrogenocarbonate and brine. After drying over magnesium sulfate and concentration in vacuum, the residue was purified by flash chromatography (EtOAc/cyclohexane 0.5:9.5 to 1:9) to afford **12a** as a yellow oil (2.56 g, 87%). $[\alpha]_D^{24}$ –27 (c = 1.9, $CHCl_3$). IR ν_{max} (cm^{-1}) 2946, 2869, 2362, 2341,

1781, 1729, 1465, 1096, 1035, 886. ^{1}H NMR (400 MHz, $CDCl_3$) δ 7.32-7.23 (m, 5H, Ph), 5.78 (s, 2H, CH_2Ph), 5.38 (s, 1H, H1), 4.92 (d, *J* = 9.6 Hz, 1H, H3), 4.33 (dq, *J* = 1.8 Hz, 7.3 Hz, 2H, Et), 4.18 (dd, *J* = 3.6 Hz, 13.3 Hz, 1H, H5), 4.12 (dd, *J* = 2.8 Hz, 12.8 Hz, 1H, H5'), 4.01 (dt, *J* = 3.2 Hz, 9.6 Hz, 1H, H4), 1.30 (t, *J* = 6.9 Hz, 3H, Et), 1.15-1.03 (m, 16H, -$CH(CH_3)_2$). ^{13}C NMR (100 MHz, $CDCl_3$) δ 209.2 (C2), 157.8 (-CO_2Et), 146.2 (triazole), 134.7 (Cq Ph), 128.9, 128.5, 128.0 (Ph), 126.5 (triazole), 80.5 (C4), 74.2, 74.1 (C1, C3), 62.6 (C5), 62.1 (Et), 54.5 (-CH_2Ph), 19.8 (CH_3), 17.7-17.2 (-$CH(CH_3)_2$), 14.3-12.8 (-$CH(CH_3)_2$, Et). HRMS calcd for $C_{29}H_{45}N_3O_7Si_2$: 603.2796; Found: 603.2782.

Ethyl 1-benzyl-5-(3',5'-*O*-(tetraisopropyldisiloxane-1,3-diyl)-β-D-erythropentofuran-2-ulosyl)-1,2,3-triazole-4-carboxylate (12b)

The same procedure as described for **12a**, starting from **11b** (5 g, 8.25 mmol) and Dess-Martin periodinane (5.2 g, 16.5 mmol) in DCM (150 mL) led to **12b** as a yellow oil (2.17 g, 44%). $[\alpha]_D^{25}$ –7 (c = 1, $CHCl_3$). IR ν_{max} (cm^{-1}) 2944, 2867, 2360, 2341, 1723, 1498, 1247, 1042. ^{1}H NMR (400 MHz, $CDCl_3$) δ 7.22-6.97 (m, 5H, Ph), 5.67 (d, *J* = 15.6 Hz, 1H, CH_2Ph), 5.61 (d, *J* = 15.6 Hz, 1H, CH_2Ph), 5.34 (s, 1H, H1), 4.55 (d, *J* = 9.6 Hz, 1H, H3), 4.31 (q, *J* = 7.3 Hz, 2H, Et), 4.06-4.01 (m, 2H, H5, H5'), 3.86-3.84 (m, 1H, H4), 1.32 (t, *J* = 6.9 Hz, 3H, Et), 1.03-0.94 (m, 28H, -$CH(CH_3)_2$). ^{13}C NMR (100 MHz, $CDCl_3$) δ 206.5 (C2), 160.9 (-CO_2Et), 138.3 (triazole), 134.7 (triazole), 134.3 (Cq Ph), 129.1, 128.6, 126.6 (Ph), 81.3 (C4), 73.5 (C3), 72.4 (C1), 62.1 (C5), 61.7 (Et), 53.0 (-CH_2Ph), 17.3-16.8 (-$CH(CH_3)_2$), 14.3-12.8 (-$CH(CH_3)_2$, Et). HRMS calcd for $C_{29}H_{46}N_3O_7Si_2$: 604.2874. Found: 604.2875.

Ethyl 1-benzyl-4-(2'-*C*-methyl-3',5'-*O*-(tetraisopropyldisiloxane-1,3-diyl)-β-D-ribofuranosyl)-1,2,3-triazole-5-carboxylate (13a)

To a solution of compound **12a** (200 mg, 0.33 mmol) in DCM (10 mL) at 0 °C was added dropwise under argon a 3 M ethereal solution of methylmagnesium bromide (0.33 mL, 1 mmol). After stirring at rt for 30 min sat. sodium hydrogenocarbonate was added and the solution was extracted with EtOAc. The organic phase was dried over magnesium sulfate and concentrated in vacuum. The residue was purified by flash chromatography (EtOAc/cyclohexane 0.5:9.5) to afford **13a** as an oil (38 mg, 19%). $[\alpha]_D^{25}$ –30 (c = 1.4, $CHCl_3$). IR ν_{max} (cm^{-1}) 3734, 2926, 2868, 2360, 2342, 1728, 1465, 1257, 1036 cm^{-1}. ^1H NMR (400 MHz, $CDCl_3$) 7.33-7.22 (m, 5H, Ph), 5.87 (d, *J* = 4.1 Hz, 2H, CH_2Ph), 5.53 (s, 1H, H1'), 4.54 (d, *J* = 6.9 Hz, 1H, H3'), 4.34 (q, *J* = 7.3 Hz, 2H, $CO_2CH_2CH_3$), 4.24-4.17 (m, 1H, H5'a), 4.09-4.04 (m, 2H, H4' et H5'b), 1.34 (t, J = 7.3 Hz, 3H, $CO_2CH_2CH_3$), 1.24-1.01 (m, 16H, - $CH(CH_3)_2$), 1.00 ppm (s, 3H, CH_3). ^{13}C NMR (100 MHz, $CDCl_3$) δ 158.4 (-CO_2Et), 149.3 (-triazole), 134.9 (Ph), 128.8, 128.4, 127.8 (Ph), 125.2 (-CCO_2Et triazole), 82.6 (C4'), 80.2 (C2'), 79.2 (C1'), 79.0 (C3'), 64.3 (C5'), 62.2 (-$CO_2CH_2CH_3$), 53.9 (-CH_2Ph), 22.2 (CH_3), 17.8-17.2 (-$CH(CH_3)_2$), 14.1-12.8 ppm (-$CH(CH_3)_2$ and -CH_2CH_3). HRMS cald pour $C_{30}H_{49}N_3O_7Si_2$: 619.3109; Found : 619.3087.

Ethyl 1-benzyl-4-(2'-*C*-methyl-3',5'-*O*-(tetraisopropyldisiloxane-1,3-diyl)-β-D-arabofuranosyl)-1,2,3-triazole-5-carboxylate (13b)

To a solution of compound **12a** (323 mg, 0.54 mmol) in DCM (15 mL) at 0 °C was added dropwise under argon a 2.4 M hexane solution of trimethyl aluminium (0.23 mL, 0.54 mmol). After stirring at rt for 1 h sat. sodium hydrogenocarbonate was added and the solution was extracted with EtOAc. The organic phase was dried over

magnesium sulfate and concentrated in vacuum. The residue was purified by flash chromatography (EtOAc/cyclohexane 0.5:9.5) to afford **13b** as an oil (290 mg, 87%). $[\alpha]_D^{25}$ –34 (c = 2, $CHCl_3$). IR ν_{max} (cm^{-1}) 3443, 2944, 2868, 2360, 2344, 1730, 1465, 1255, 1105, 1038. ^1H NMR (400 MHz, $CDCl_3$) δ 7.31-7.27 (m, 5H, Ph), 5.84 (s, 2H, CH_2Ph), 5.08 (s, 1H, H1), 4.34 (dq, *J* = 2.3Hz, 6.9 Hz, 2H, Et), 4.30 (d, *J* = 2.3 Hz, 1H, H3), 4.11-4.08 (m, 1H, H5), 3.95-3.92 (m, 2H, H4, H5'), 1.34 (t, *J* = 7.3 Hz, 3H, Et), 1.28 (s, 3H, CH_3), 1.11-1.01 (m, 28H, -$CH(CH_3)_2$). ^{13}C NMR (100 MHz, $CDCl_3$) δ 158.3 (-CO_2Et), 146.7 (triazole), 134.7 (Cq Ph), 128.9, 128.6, 128.1 (Ph), 127.0 (triazole), 87.2 (C4), 81.5, 81.6 (C2, C3), 76.8 (C1), 60.6 (C5), 57.8 (Et), 49.6 (-CH_2Ph), 19.8 (CH_3), 17.9-17.3 (-$CH(CH_3)_2$), 14.1-12.9 (-$CH(CH_3)_2$, Et). HRMS calcd for $C_{30}H_{49}N_3O_7Si_2$: 619.3109; Found: 619.3115.

Ethyl 1-benzyl-4-(2'-deoxy-2'-fluoro-2'-methyl-3',5'-*O*-(tetra isopropyldisiloxane-1,3-diyl)-β-D-ribofuranosyl)-1,2,3-triazole -5-carboxylate (14), ethyl 1-benzyl-4-(2'-deoxy-2'-methylene-3',5'-*O*-(tetraisopropyldisiloxane-1,3-diyl)-β-D-ribo furanosyl)-1,2,3-triazole-5-carboxylate (15) and ethyl 1-benzyl-4-(2'-deoxy-2'-methylyl-3',5'-*O*-(tetra isopropyldisiloxane-1,3-diyl)-β-D-ribo furan-2'-enosyl)-1,2,3-triazole-5-carboxylate (16)

To a solution of compound **13b** (300 mg, 0.48 mmol) in anhydrous DCM (10 mL) at −20 °C was added under argon pyridine (0.24 mL, 3 mmol). Diethylaminosulfur trifluoride (0.68 mL, 5.2 mmol) was then introduced dropwise. The mixture was stirred at −20 °C for 1 h, then sat. sodium hydrogenocarbonate was added and the solution was extracted with EtOAc. The organic phase was dried over magnesium sulfate and concentrated in vacuum. The residue was purified by flash chromatography (EtOAc/cyclohexane, 0.1:9.9 to 0.5:9.5) to afford an oil corresponding to a 6:4

mixture of **14** (72 mg, 24%), **15** (46 mg, 16%) and **16** (57 mg, 20%, oil). **14**: ^{1}H NMR (400 MHz, $CDCl_3$) δ 7.28-7.15 (m, 5H, Ph), 5.79 (d, *J* = 5.5 Hz, 2H, CH_2Ph), 5.62 (d, *J* = 25 Hz, 1H, H1), 4.61 (dd, *J* = 10 Hz, 25 Hz, 1H, H3), 4.28 (q, *J* = 7.3 Hz, 2H, $CO_2CH_2CH_3$), 4.05-3.96 (m, 3H, H4, H5, H5'), 1.26 (t, *J* = 7.3 Hz, 3H, Et), 1.25 (d, *J* = 5 Hz, 3H, CH_3), 1.11-0.99 (m, 28, -CH(CH_3)$_2$). ^{13}C NMR (100 MHz, $CDCl_3$): δ 158.4 (-CO_2Et), 148.5 (d, *J* = 15 Hz, triazole), 135.0 (Cq Ph), 128.8-127.8 (Ph), 125.6 (triazole), 101.8 (d, *J* = 184 Hz, C2), 80.7 (C4), 78.2 (d, *J* = 30 Hz, C1), 75.4 (d, *J* = 16 Hz, C3), 62.3 (Et), 61.8 (C5), 53.9 (-CH_2Ph), 18.1 (d, *J* = 27 Hz, CH_3), 17.8-17.1 (-CH(CH_3)$_2$), 14.0 (Et), 13.7-12.7 (-CH(CH_3)$_2$). ^{19}F NMR (376.2 MHz, $CDCl_3$)**:** δ –156.3 (m). HRMS calcd for $C_{30}H_{49}FN_3O_6Si_2$: 622.3144; Found: 622.3115. **15**: ^{1}H NMR (400 MHz, $CDCl_3$) δ 7.28-7.15 (m, 5H, Ph), 5.97 (s, 1H, H1), 5.79 (d, *J* = 5.5 Hz, 2H, CH_2Ph), 5.21 (s, 1H, =CH), 5.08 (d, *J* = 8.7 Hz, 1H, H3), 4.94 (s, 1H, =CH), 4.28 (q, *J* = 7.3 Hz, 2H, Et), 4.14 (dd, *J* = 3.7 Hz, 11.9 Hz, 1H, H5), 4.05-3.96 (m, 1H, H5'), 3.72-3.66 (m, 1H, H4), 1.27 (t, *J* = 7.3 Hz, 3H, Et), 1.11-0.99 (m, 28H, -CH(CH_3)$_2$). ^{13}C NMR (100 MHz, $CDCl_3$) δ 158.5 (-CO_2Et), 151.2, 151.1 (triazole, C2), 135.1 (Cq Ph), 128.8-127.8 (Ph), 124.9 (triazole), 107.1 (C=), 83.8 (C4), 74.3 (C3), 73.0 (C1), 63.2 (C5), 62.1 (Et), 53.9 (-CH_2Ph), 17.8-17.1 (-CH(CH_3)$_2$), 14.1 (Et), 13.7-12.7 (-CH(CH_3)$_2$). HRMS calcd for $C_{30}H_{48}N_3O_6Si_2$: 602.3082; Found: 602.3092. **16**: $[\alpha]_D^{25}$ −9 (c = 0.45, $CHCl_3$). IR ν_{max} (cm^{-1}) 2927, 2867, 2359, 2342, 1729, 1465, 1258, 1097. ^{1}H NMR (400 MHz, $CDCl_3$) δ 7.32-7.26 (m, 5H, Ph), 5.85 (s, 2H, CH_2Ph), 5.18 (d, *J* = 5.5 Hz, 1H, H3'), 4.43 (ddd, *J* = 4.5 Hz, 5.5 Hz, 11 Hz, 1H, H4'), 4.26 (q, *J* = 7.3 Hz, 2H, $CO_2CH_2CH_3$), 4.34 (dd, *J* = 4.5 Hz, 11 Hz, 1H, H5'a), 3.76 (dd, *J* = 11 Hz, 11 Hz, 1H, H5'b), 1.74 (s, 3H, CH_3), 1.28 (t, *J* = 7.3 Hz, 3H, $CO_2CH_2CH_3$), 1.11-1.00 (m, 16H, -CH(CH_3)$_2$). ^{13}C NMR (100 MHz, $CDCl_3$) δ 158.8 (-CO_2Et), 141.5 (C1'), 141.2 (triazole), 134.8 (Ph), 128.9, 128.5, 128.1 (Ph), 126.4 (triazole), 113.5 (C2'), 87.4

(C4’), 81.9 (C3’), 64.6 (C5’), 62.1 (CH_2CH_3), 53.8 (-CH2Ph), 17.7-16.9 (-CH(CH_3)$_2$), 14.0 (CH_2CH_3), 13.7-12.5 (-CH(CH_3)$_2$), 9.8 (CH_3). HRMS calcd for $C_{30}H_{47}N_3O_6Si_2$: 601.3003; Found: 601.3007.

Ethyl 1-benzyl-4-(2’-deoxy-2’-fluoro-2’-methyl-β-D-ribofuranosyl)-1,2,3-triazole-5-carboxylate (17) and ethyl 1-benzyl-4-(2’-deoxy-2’-methylene-β-D-ribofuranosyl)-1,2,3-triazole-5-carboxylate (18)

To a solution of the unseparated mixture of compounds **14** and **15** (326 mg) in anhydrous THF were added glacial acetic acid (72 μL, 1.26 mmol) followed by tetrabutylammonium fluoride (1.6 mL, 1.63 mmol). The mixture was stirred for 15 min at rt and then concentrated in vacuum. The residue was purified by flash chromatography (EtOAc/cyclohexane, 0.1:9.9 to 4:6 to 6:4) to afford **17** (125 mg) and **18** (79 mg) in quantitative yield. **17:** $[\alpha]_D^{25}$ +15 (c = 1, MeOH). IR ν_{max} (cm^{-1}) 3385, 2939, 1728, 1497, 1324, 1107, 1010, 855. ^1H NMR (400 MHz, $CDCl_3$) δ 7.33-7.22 (m, 5H, Ph), 5.87 (d, *J* = 6.0 Hz, 2H, CH_2Ph), 5.73 (d, *J* = 22 Hz, 1H, H1), 5.73 (dd, *J* = 8.7 Hz, 23.8 Hz, 1H, H3), 4.42-4.34 (m, 2H, Et), 4.13 (d, *J* = 12.4 Hz, 1H, H5), 4.10 (d, *J* = 8.7 Hz, 1H, H4), 3.86 (d, *J* = 12.4 Hz, 1H, H5’), 1.36 (dt, *J* = 1.8 Hz, 7.3 Hz, 3H, Et), 1.16 (d, *J* = 22 Hz, 3H, CH_3). ^{13}C NMR (100 MHz, $CDCl_3$) δ 157.9 (-CO_2Et), 149.7 (d, *J* = 16.2 Hz, triazole), 134.6 (Cq Ph), 128.9, 128.7, 127.9 (Ph), 124.8 (triazole), 102.7 (d, *J* = 179 Hz, C2), 82.8 (C4), 78.7 (d, *J* = 30 Hz, C1), 72.5 (d, *J* = 18 Hz, C3), 62.6 (Et), 61.2 (C5), 54.2 (-CH_2Ph), 17.3 (d, *J* = 26 Hz, CH_3), 14.1 (Et). ^{19}F NMR (376.2 MHz, $CDCl_3$) –161.5 (m). HRMS calcd for $C_{18}H_{23}FN_3O_5$: 380.1622; Found: 380.1627. **18:** $[\alpha]_D^{25}$ +26.9 (c = 1, MeOH). IR ν_{max} (cm^{-1}) 3373, 2926, 2365, 2344, 1727, 1497, 1261, 1105. ^1H NMR (400 MHz, $CDCl_3$) δ 7.31-7.25 (m, 5H, Ph), 6.15 (s, 1H, H1), 5.86 (s, 2H, CH_2Ph), 5.31 (s, 1H, =CH), 5.05 (s, 1H, =CH), 5.02 (d,

J = 6.4 Hz, 1H, H3), 4.37 (q, *J* = 7.3 Hz, 2H, Et), 4.06 (dd, *J* = 2.3 Hz, 12.4 Hz, 1H, H5), 4.01-3.99 (m, 1H, H4), 3.84 (dd, *J* = 2.7 Hz, 12.4 Hz, 1H, H5'), 1.34 (t, *J* = 7.3 Hz, 3H, Et). ^{13}C NMR (100 MHz, $CDCl_3$) δ 158.3 (-CO_2Et), 152.4 (triazole), 151.9 (C2), 134.8 (Cq Ph), 128.9, 128.6, 128.0 (Ph), 124.2 (triazole), 109.3 (C=), 85.7 (C4), 73.8 (C1), 71.5 (C3), 62.4 (Et), 62.3 (C5), 54.2 (-CH_2Ph), 14.2 (Et). HRMS calcd for $C_{18}H_{22}N_3O_5$: 360.1559; Found: 360.1565.

1,5-*O*-diacetyl-2,3-*O*-isopropylidene-2-*C*-methyl-D-ribofuranose (5)

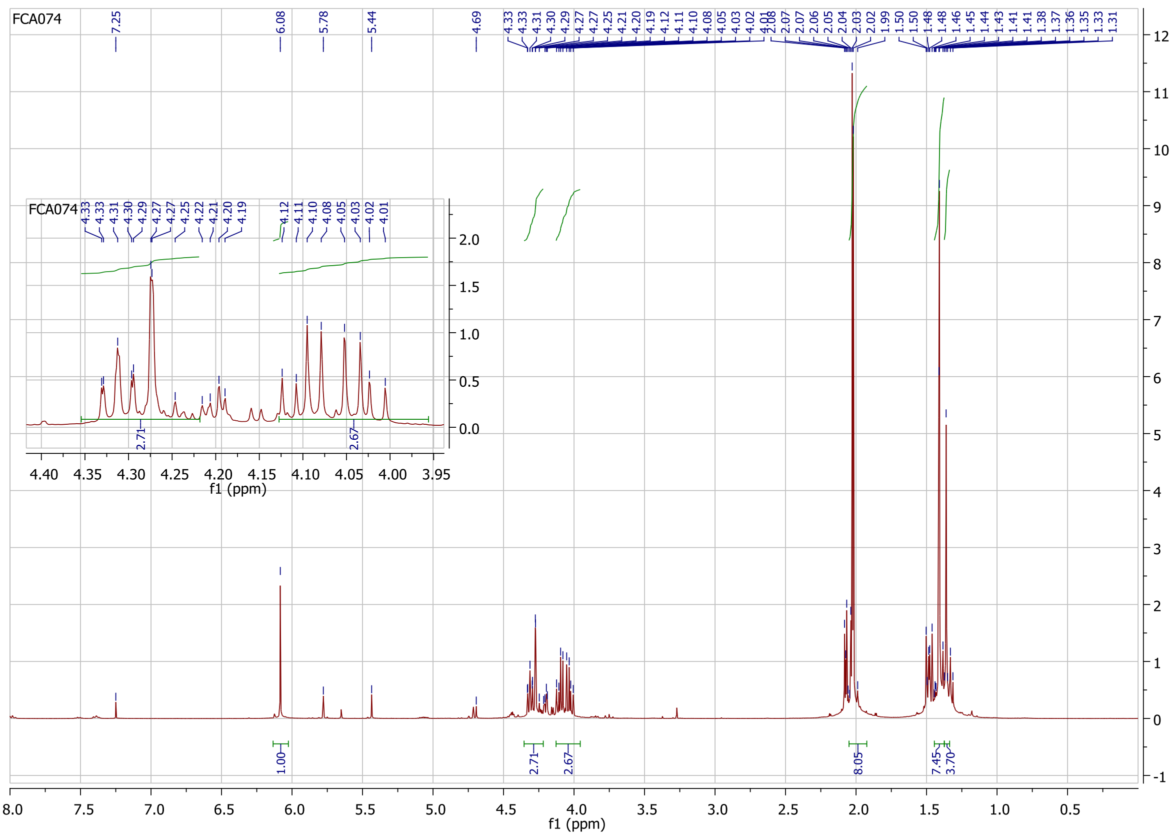

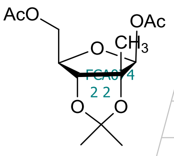

5

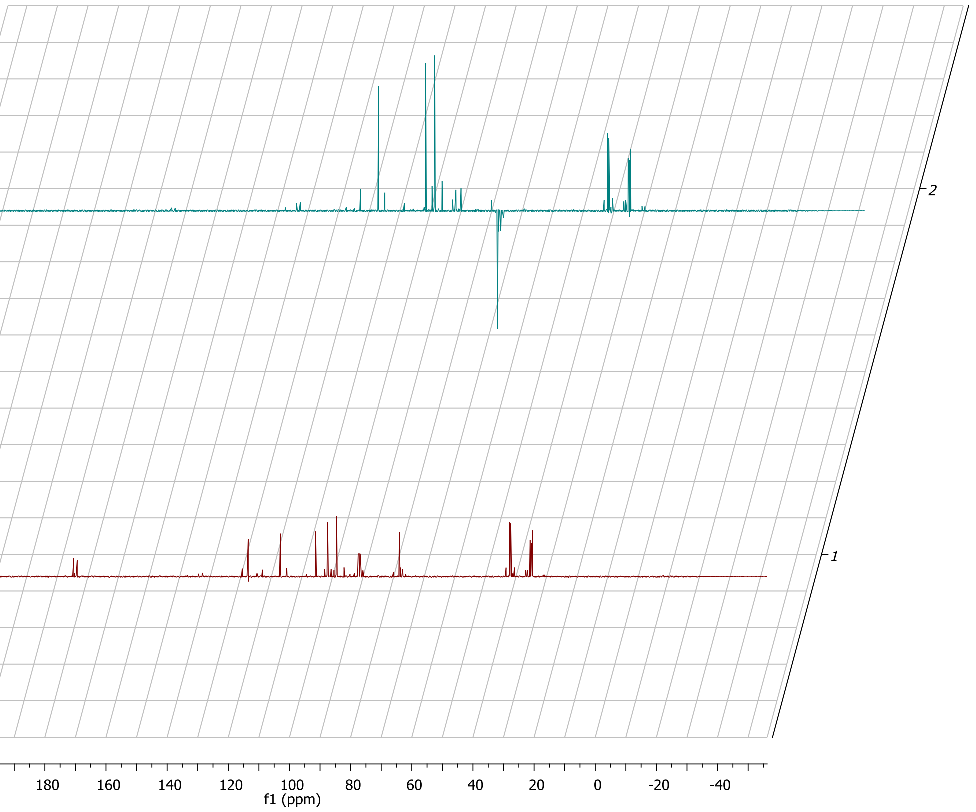

Ethyl 3-(5-*O*-acetyl-2,3-*O*-isopropylidene-2-*C*-methyl-β-D-ribofuranosyl)propiolate (6)

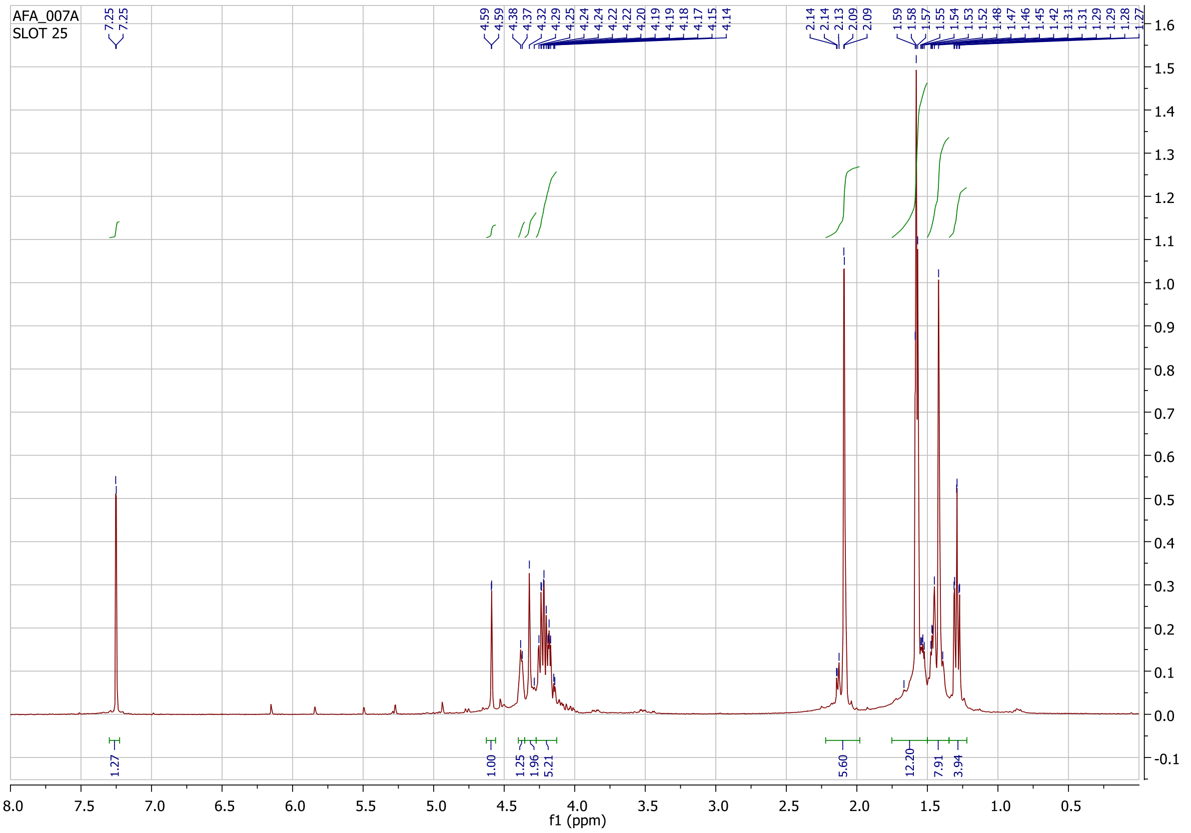

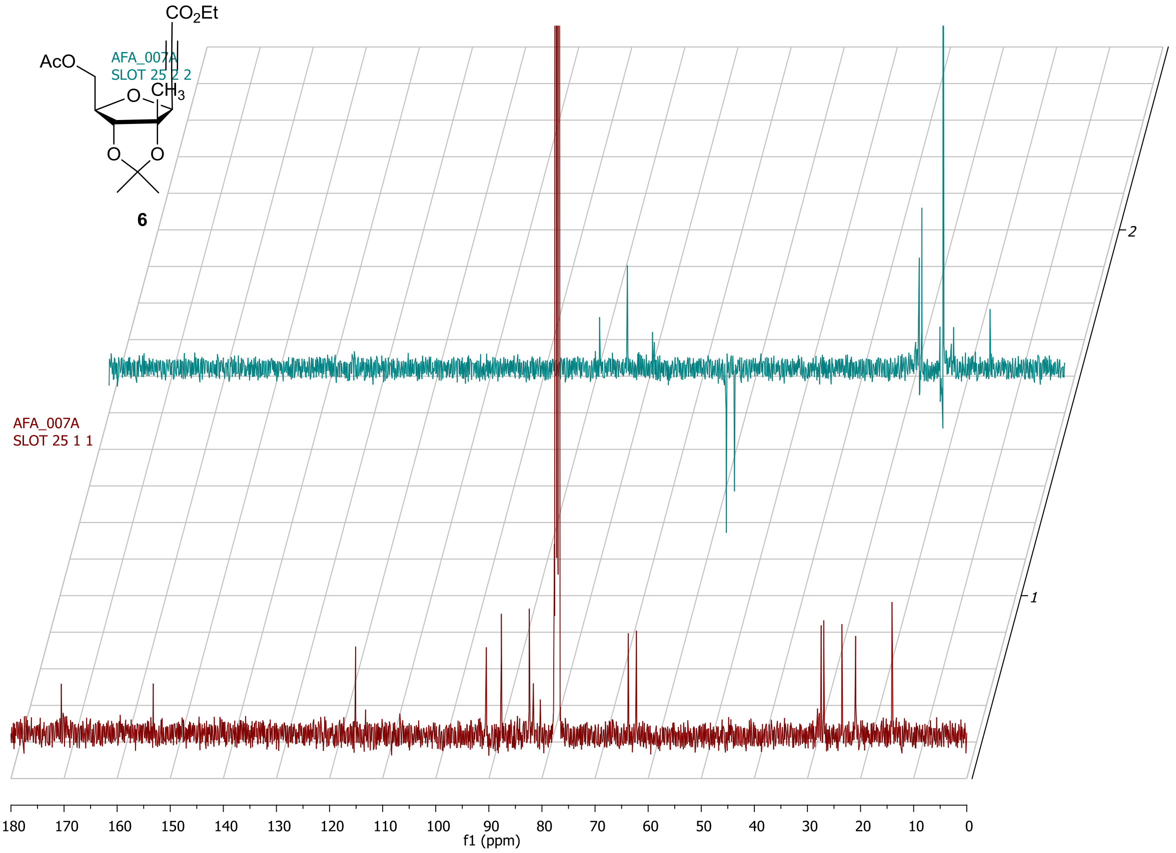

Ethyl 1-benzyl-4-(5'-*O*-acetyl-2',3'-*O*-isopropylidene-2'-*C*-methyl-β-D-ribofuranosyl)-1,2,3-triazole-5-carboxylate (7a) and ethyl 1-benzyl-4-(5'-*O*-acetyl-2',3'-*O*-isopropylidene-2'-*C*-methyl-β-D-ribofuranosyl)-1,2,3-triazole-5-carboxylate (7b)

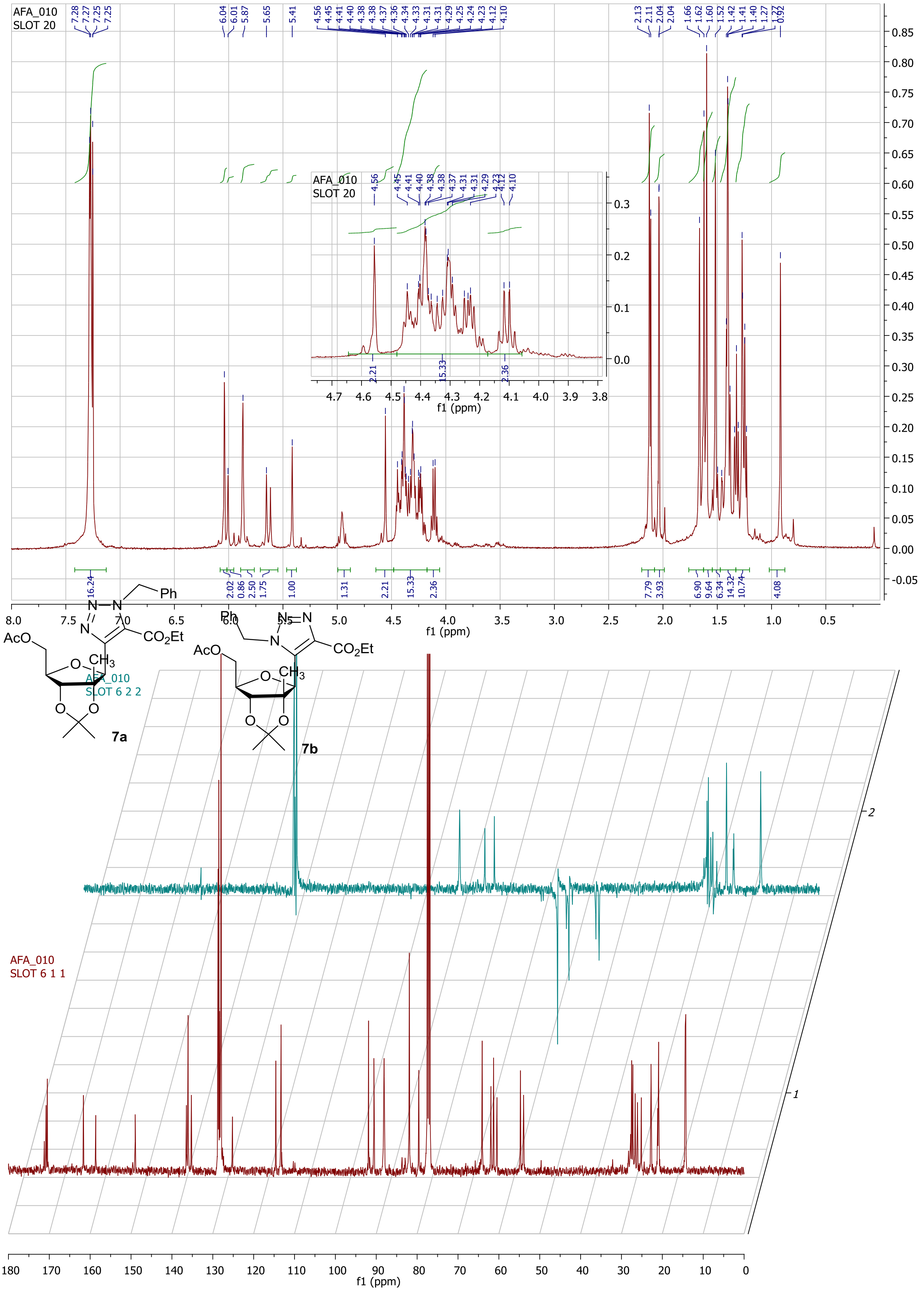

5-(2’-*C*-Methyl-β-D-ribofuranosyl)-1,2,3-triazole-4-carboxamide (2)

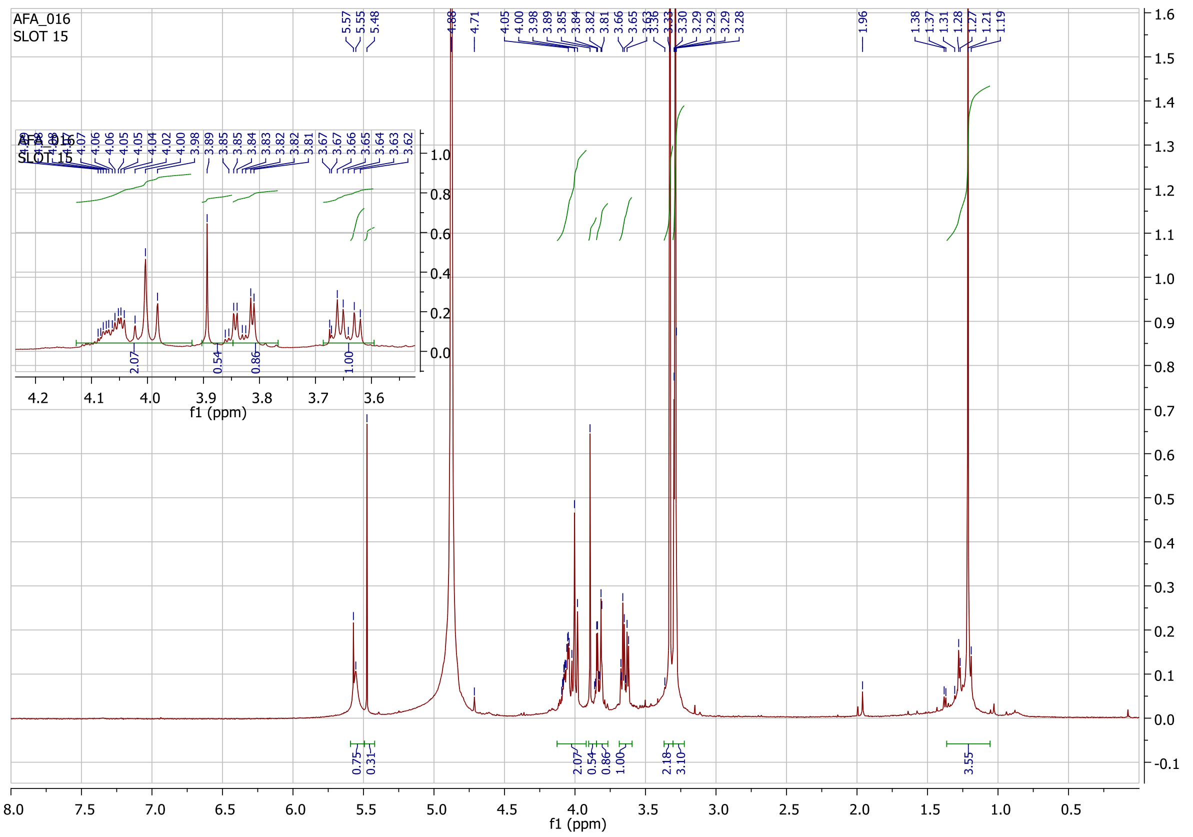

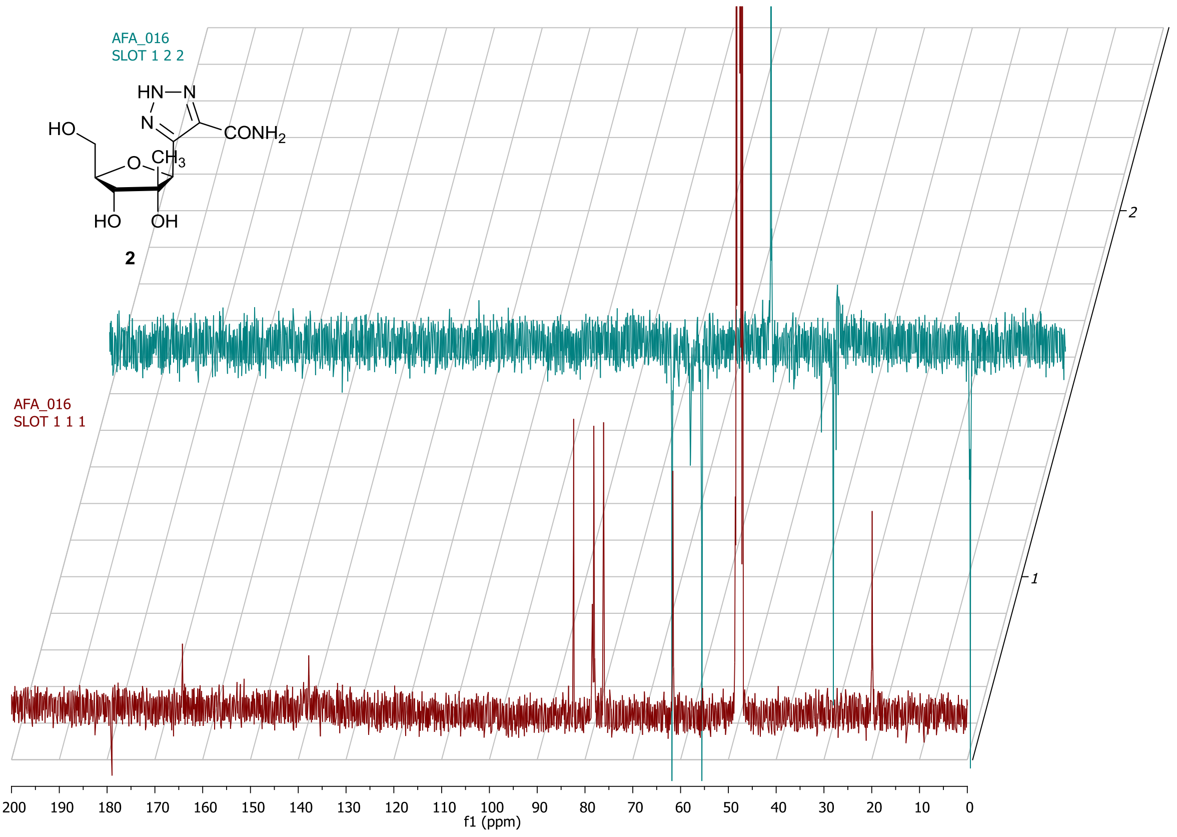

Ethyl 1-benzyl-4-(β-D-ribofuranosyl)-1,2,3-triazole-5-carboxylate (10a)

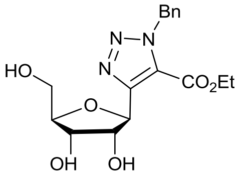

10a

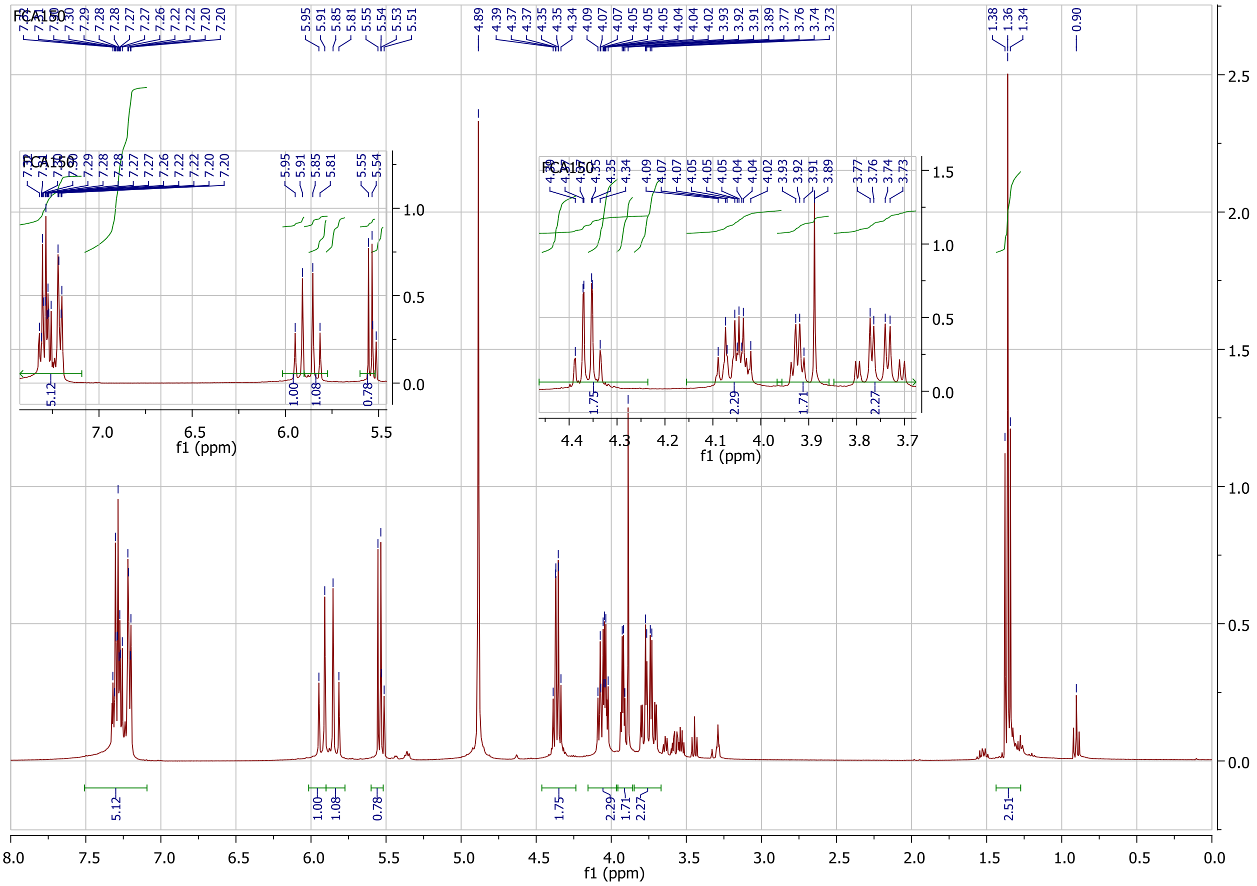

Ethyl 1-benzyl-5-(β-D-ribofuranosyl)-1,2,3-triazole-4-carboxylate (10b)

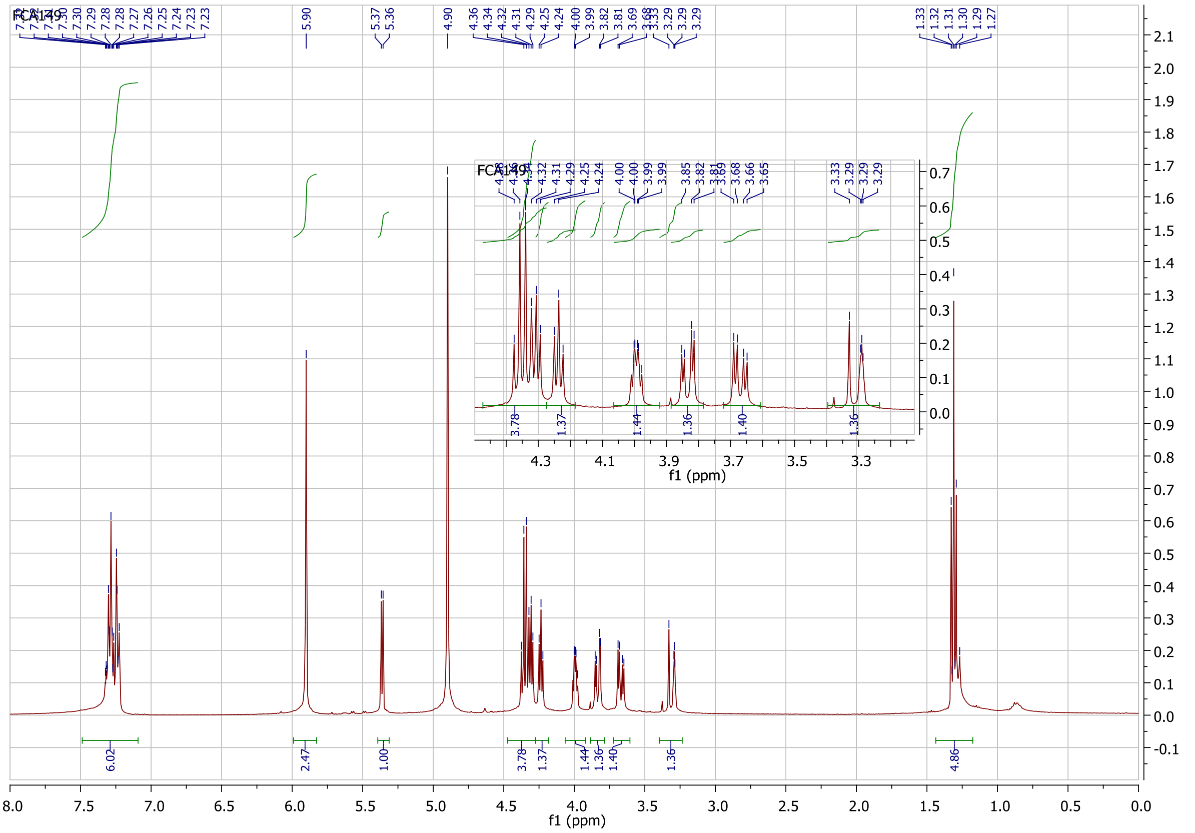

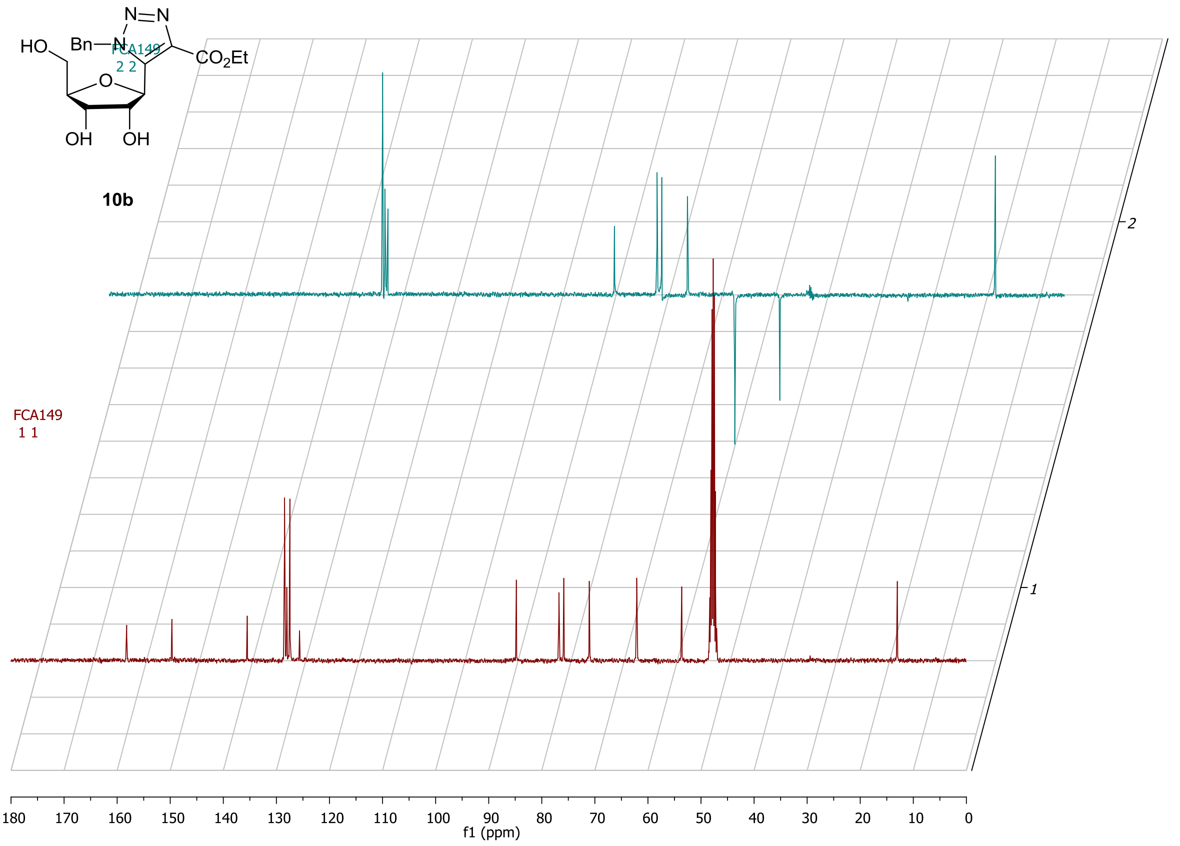

Ethyl 1-benzyl-4-(3’,5’-*O*-(tetraisopropyldisiloxane-1,3-diyl)-β-D-ribofuranosyl)-1,2,3-triazole-5-carboxylate (11a)

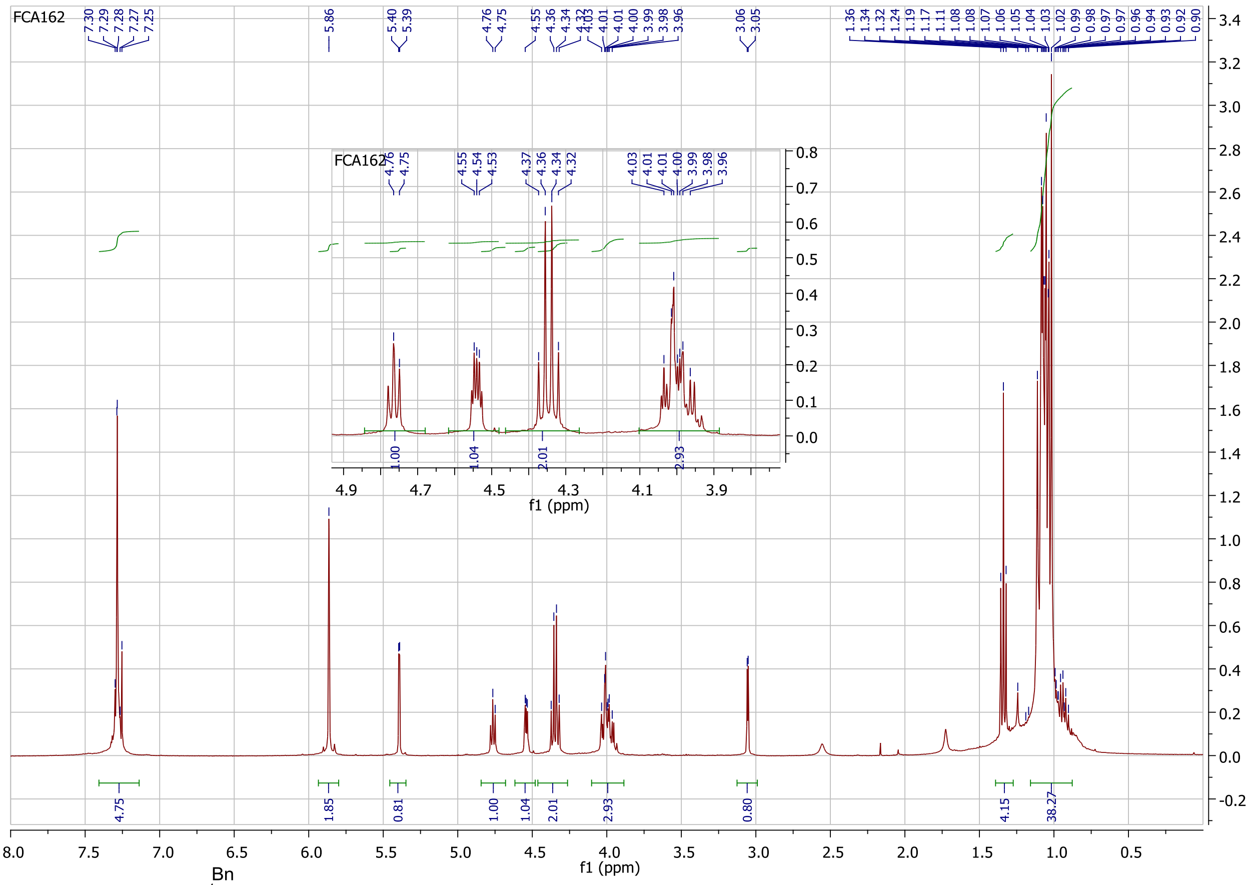

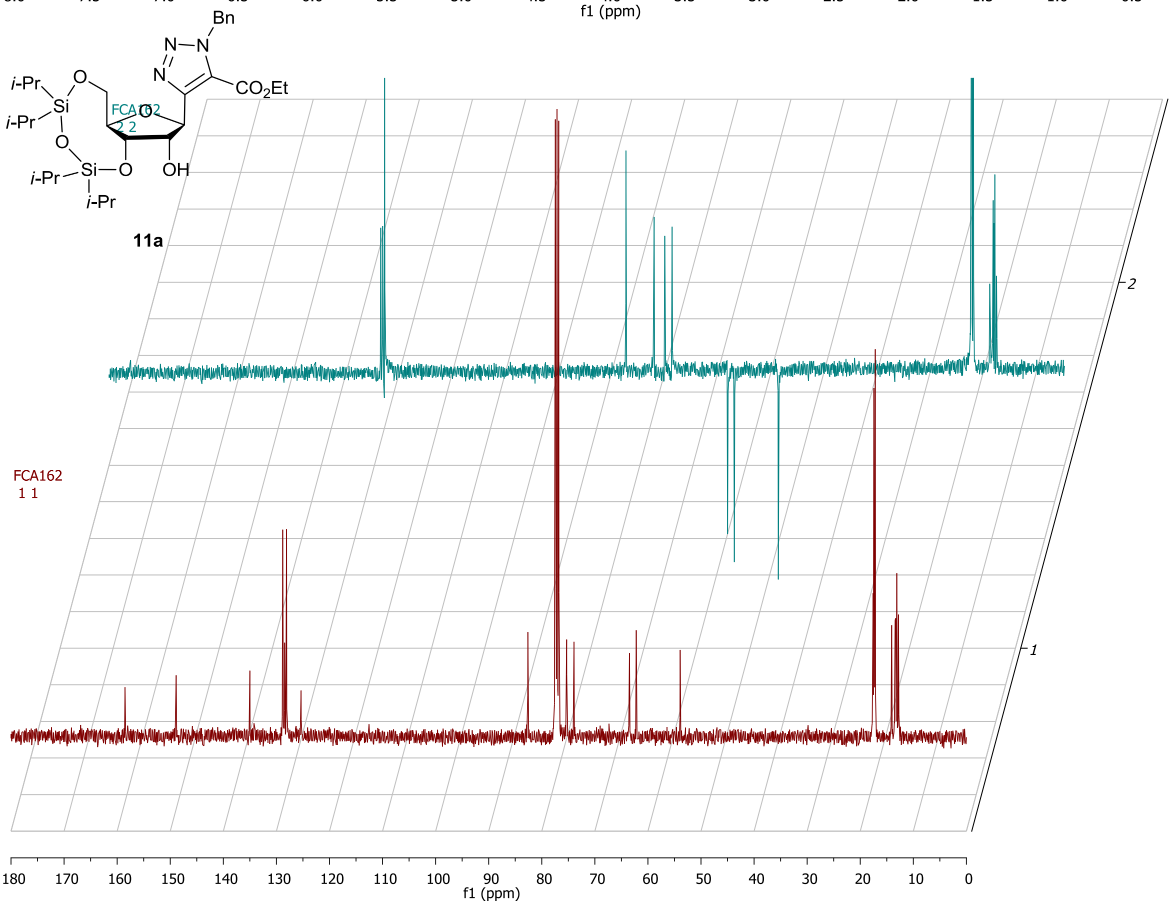

Ethyl 1-benzyl-5-(3',5'-*O*-(tetraisopropyldisiloxane-1,3-diyl)-β-D-ribofuranosyl)-1,2,3-triazole-4-carboxylate (11b)

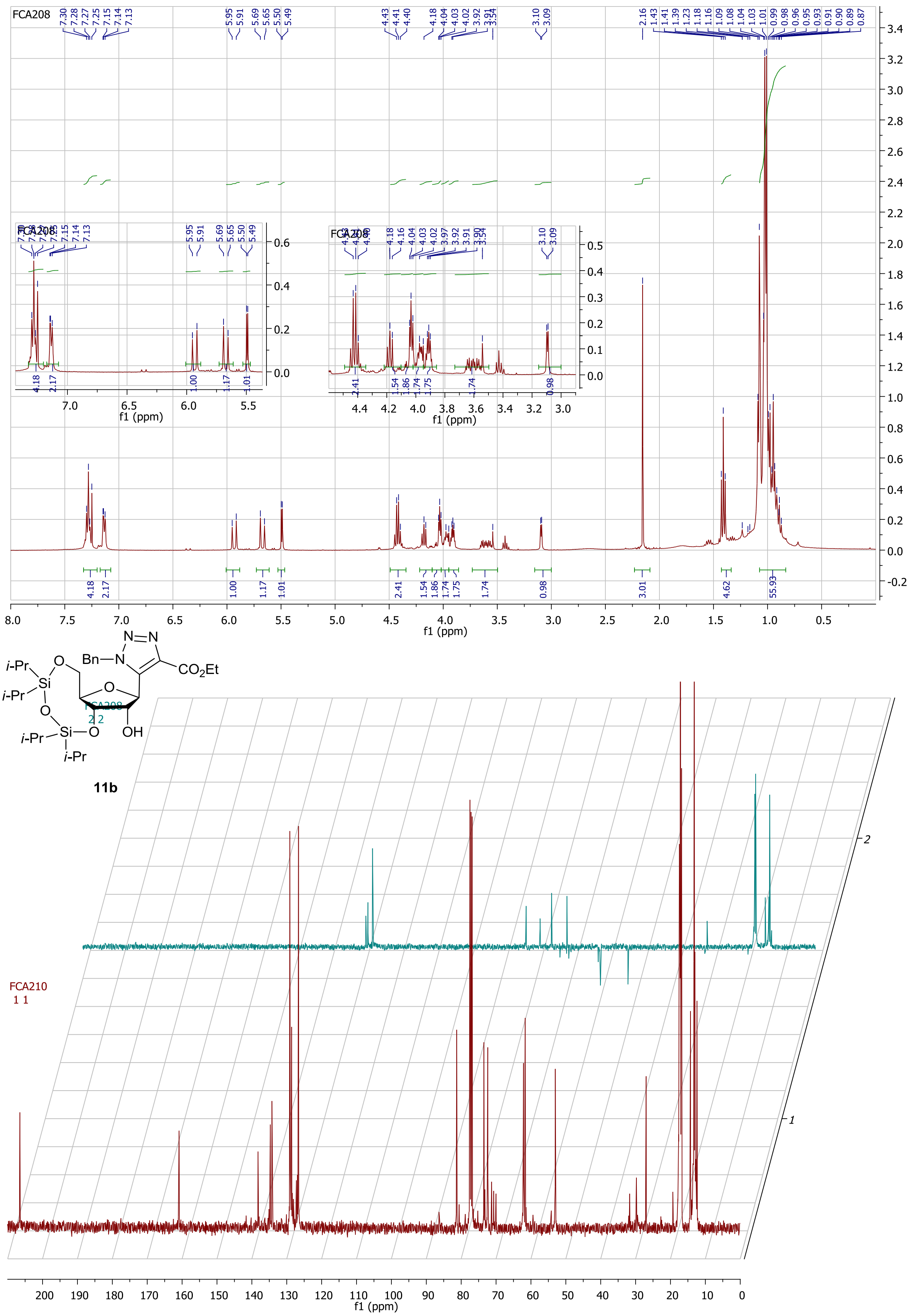

Ethyl 1-benzyl-4-(3’,5’-*O*-(tetraisopropyldisiloxane-1,3-diyl)-β-D-erythropentofuran-2-ulosyl)-1,2,3-triazole-5-carboxylate (12a)

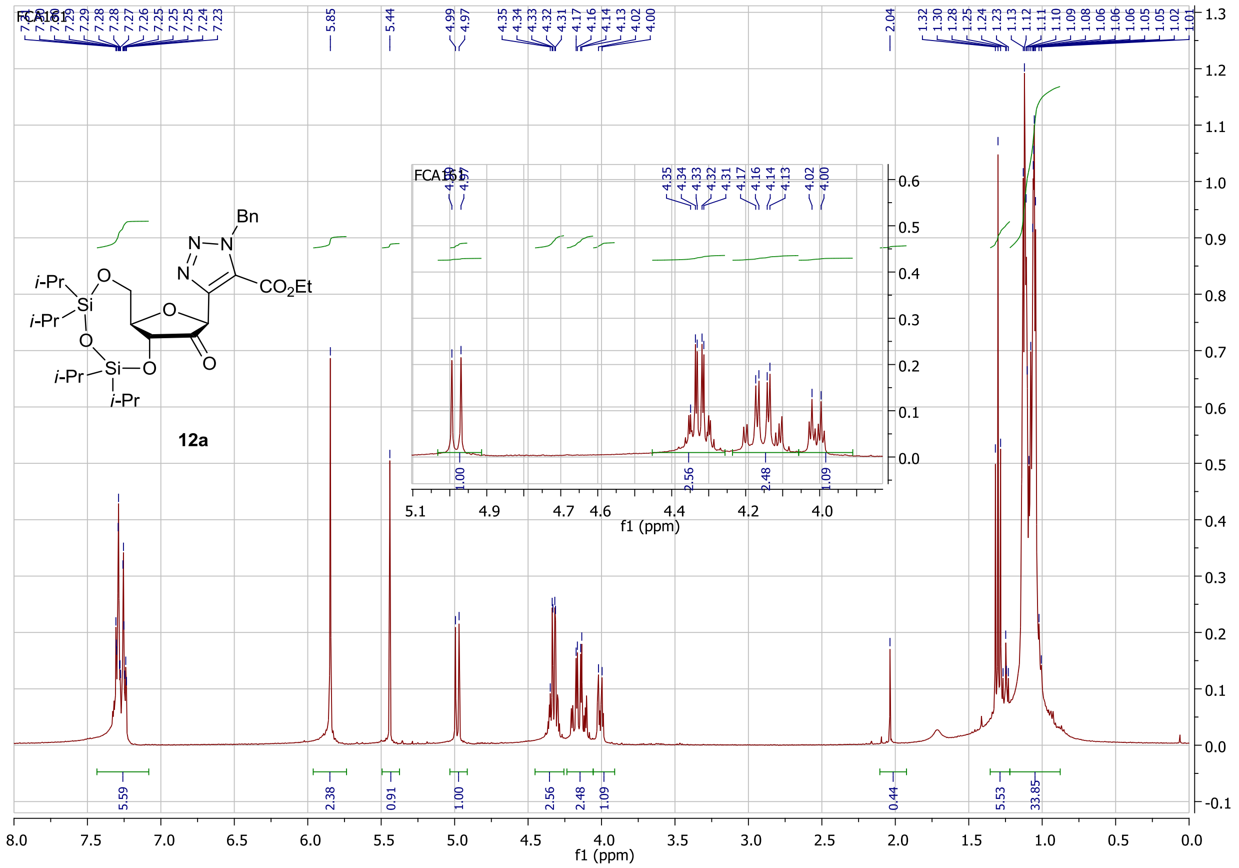

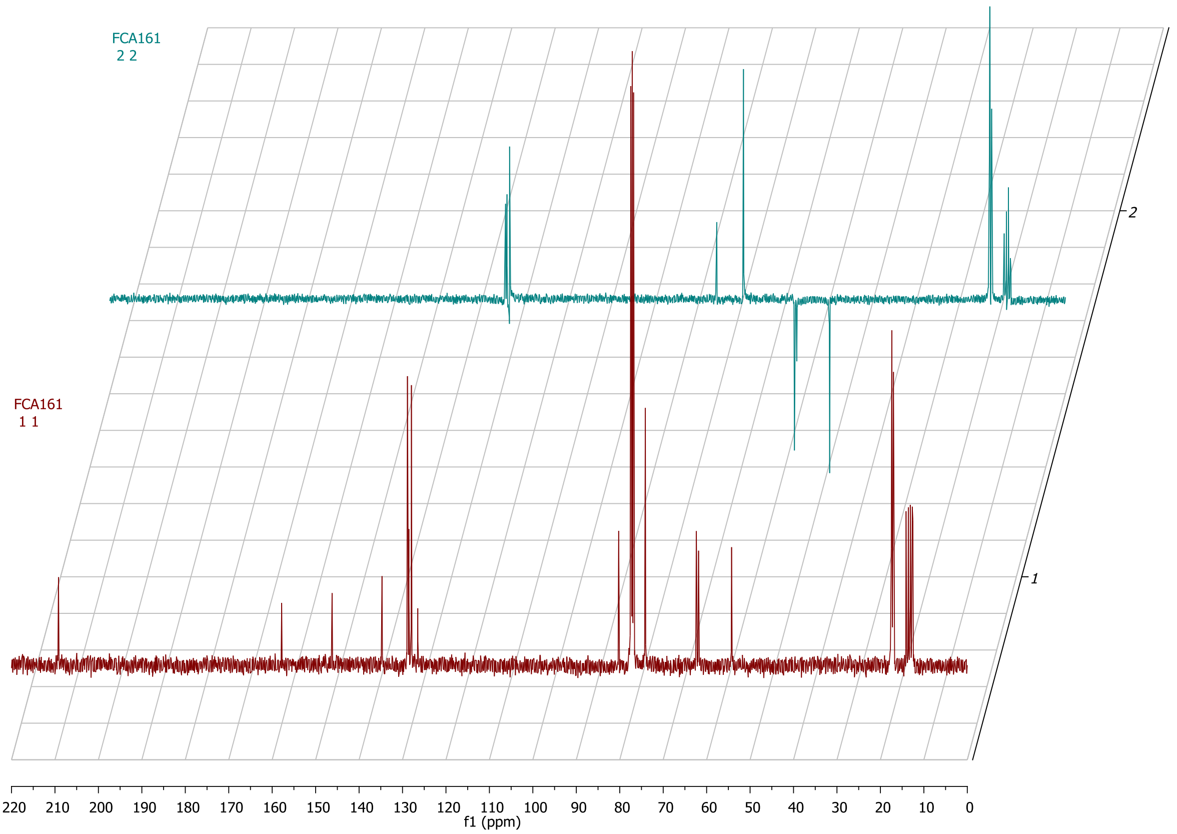

Ethyl 1-benzyl-5-(3',5'-*O*-(tetraisopropyldisiloxane-1,3-diyl)-β-D-erythropentofuran-2-ulosyl)-1,2,3-triazole-4-carboxylate (12b)

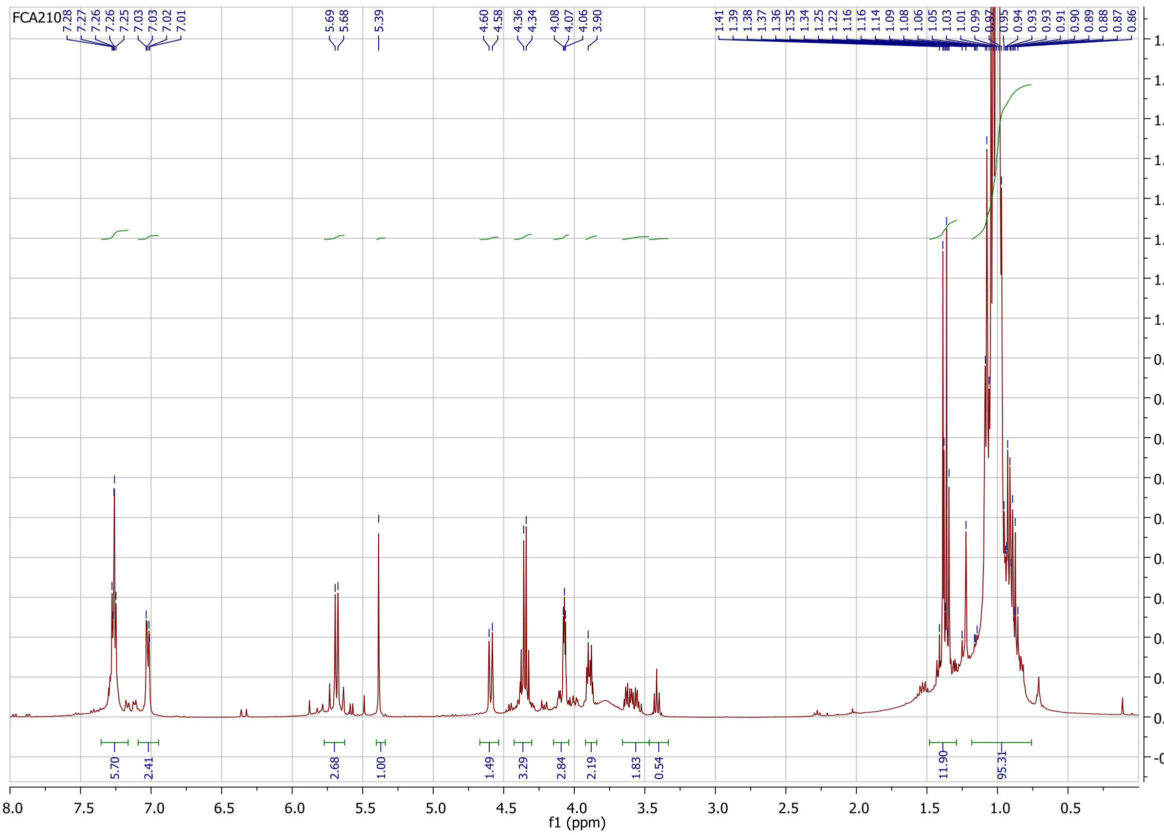

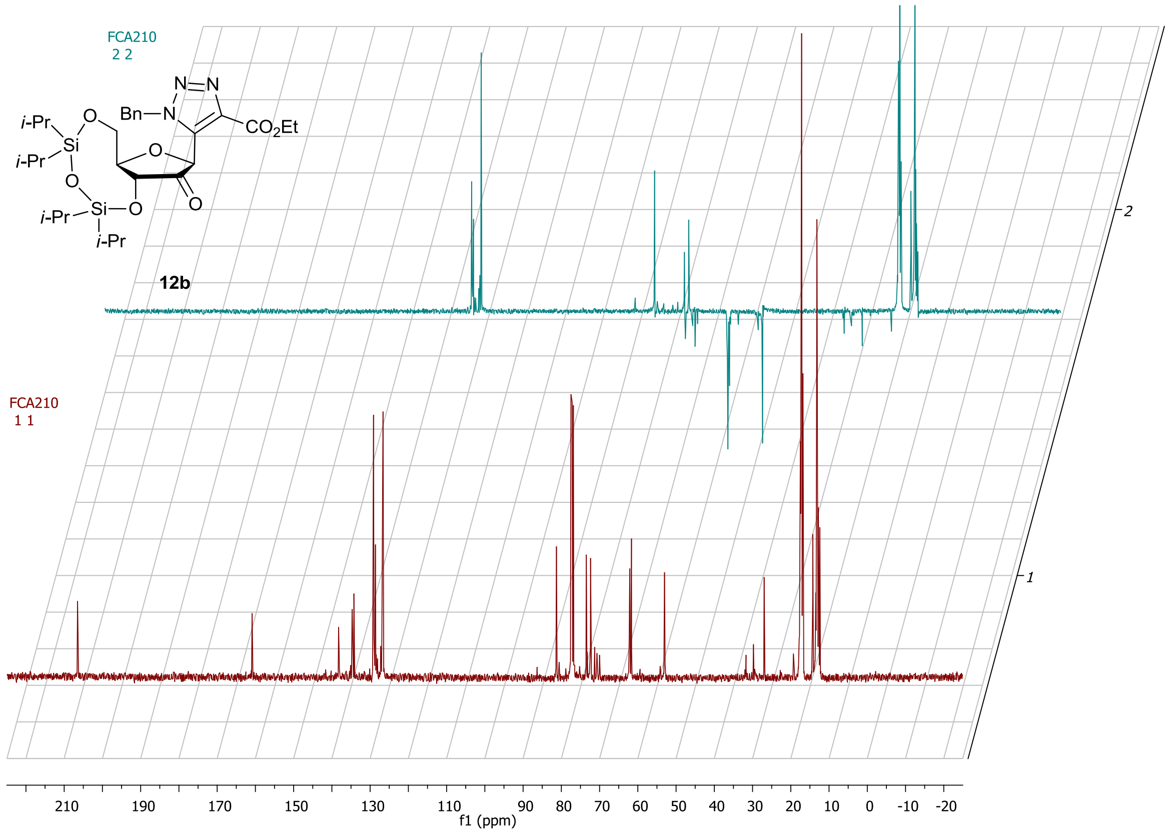

Ethyl 1-benzyl-4-(2’-*C*-methyl-3’,5’-*O*-(tetraisopropyldisiloxane-1,3-diyl)-β-D-ribofuranosyl)-1,2,3-triazole-5-carboxylate (13a)

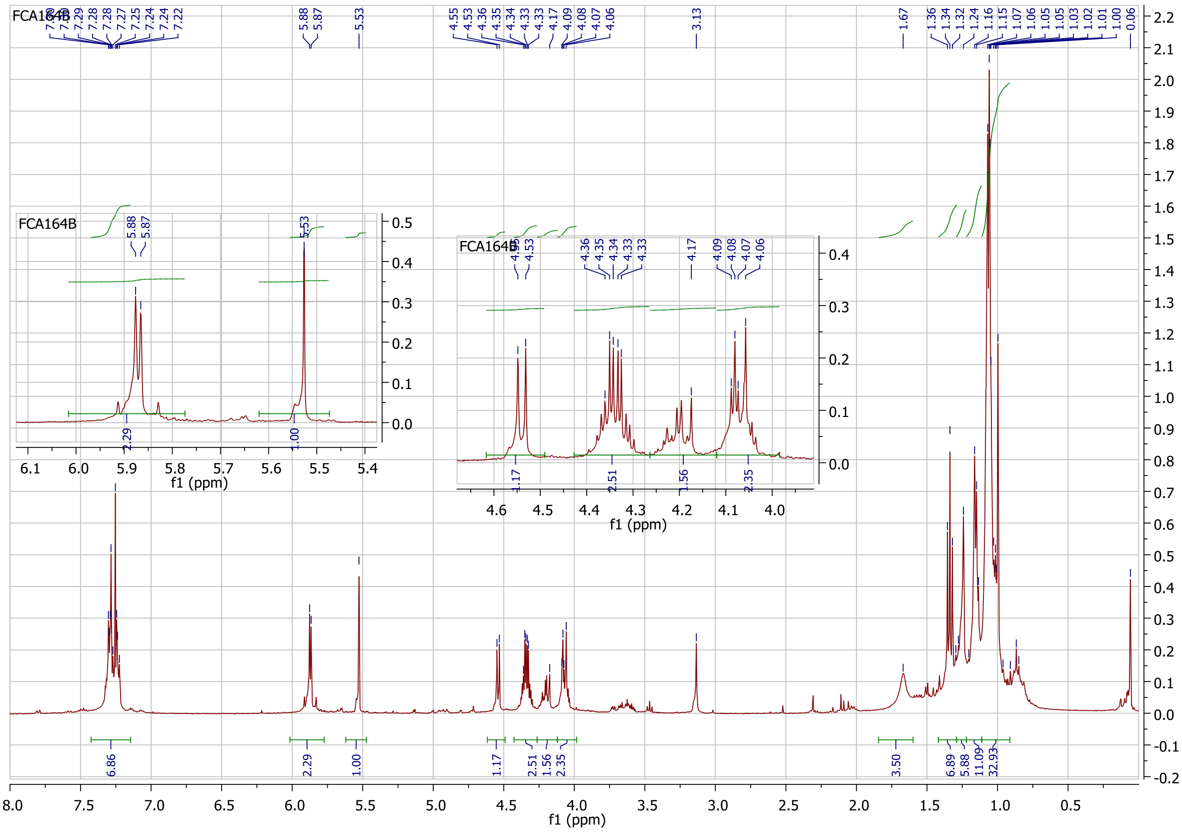

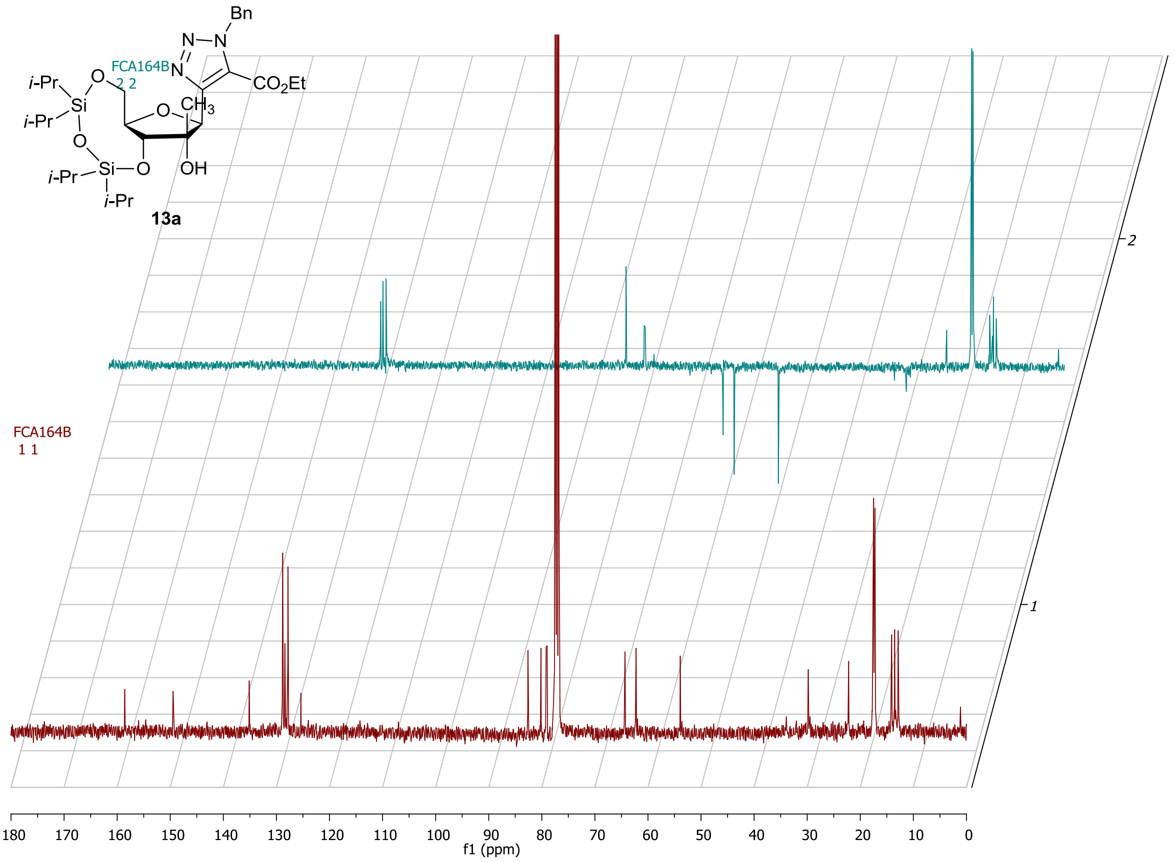

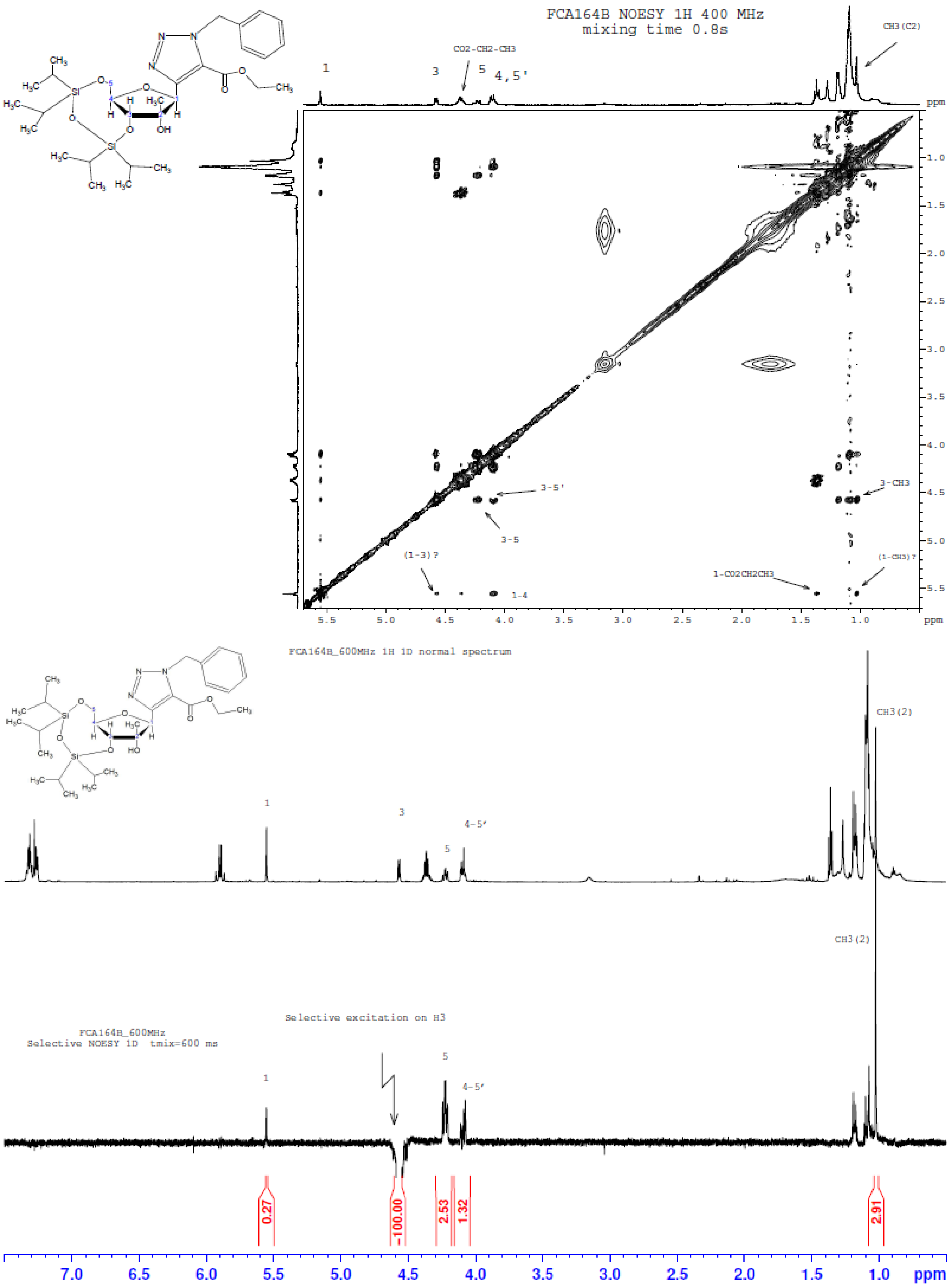
FCA164B NOESY 1H 400 MHz
mixing time 0.8s
CO2-CH2-CH3
CH3(C2)
4,5'
3-5'
3-5
(1-3)?
1-4
1-CO2CH2CH3
(1-CH3)?
3-CH3
FCA164B_600MHz 1H 1D normal spectrum
CH3(2)
4-5'
Selective excitation on H3
FCA164B_600MHz
Selective NOESY 1D tmix=600 ms
0.27
-100.00
2.53
1.32
2.91
ppm

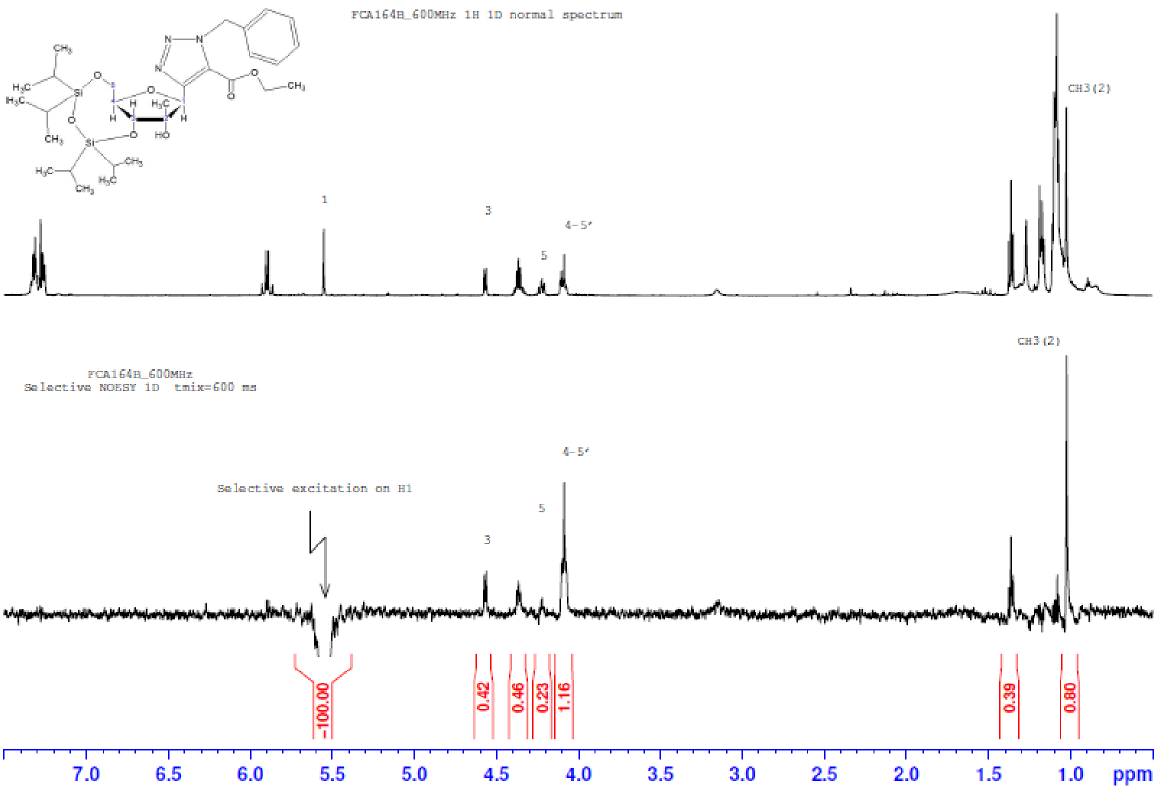
FCA164B_600MHz 1H 1D normal spectrum
CH3(2)
1
3
5
4-5'
CH3(2)
FCA164B_600MHz
Selective NOESY 1D tmix=600 ms
4-5'
Selective excitation on H1
5
3
-100.00
0.42
0.46
0.23
1.16
0.39
0.80
7.0
6.5
6.0
5.5
5.0
4.5
4.0
3.5
3.0
2.5
2.0
1.5
1.0
ppm

Ethyl 1-benzyl-4-(2'-*C*-methyl-3',5'-*O*-(tetraisopropyldisiloxane-1,3-diyl)-β-D-arabinofuranosyl)-1,2,3-triazole-5-carboxylate (13b)

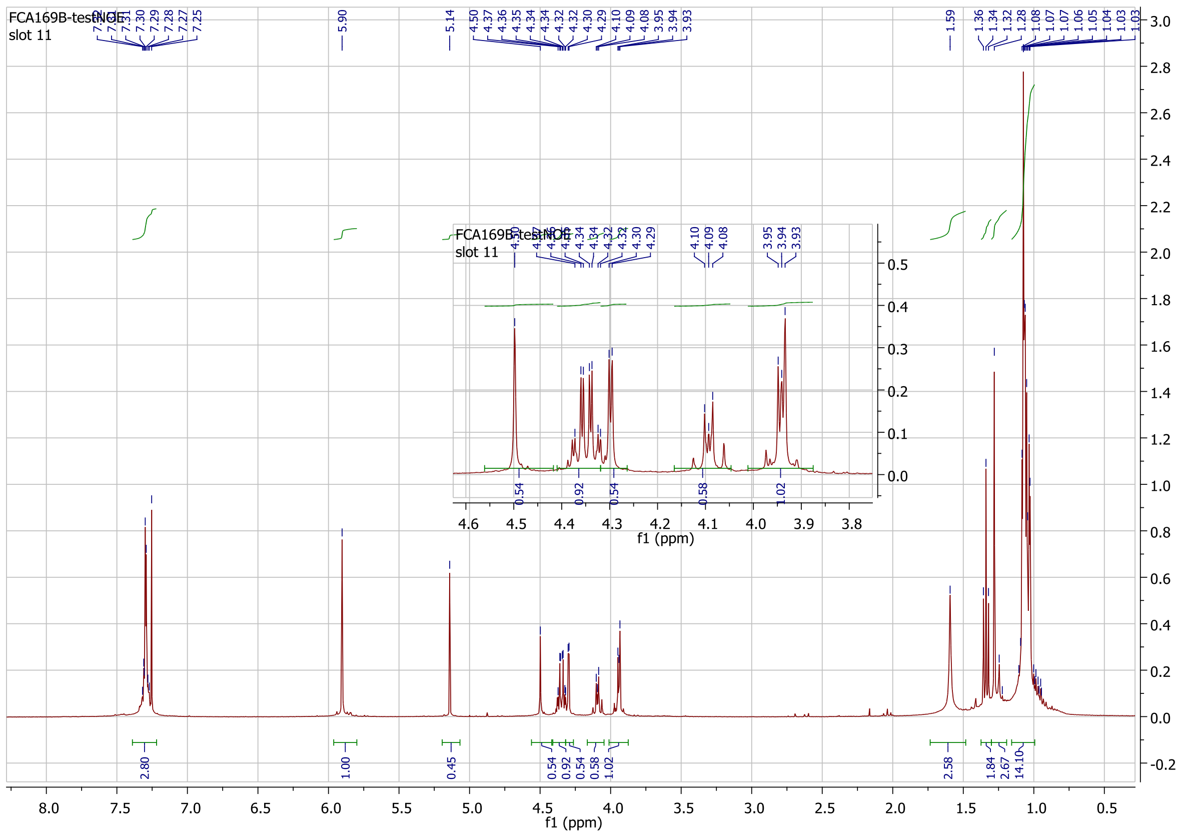

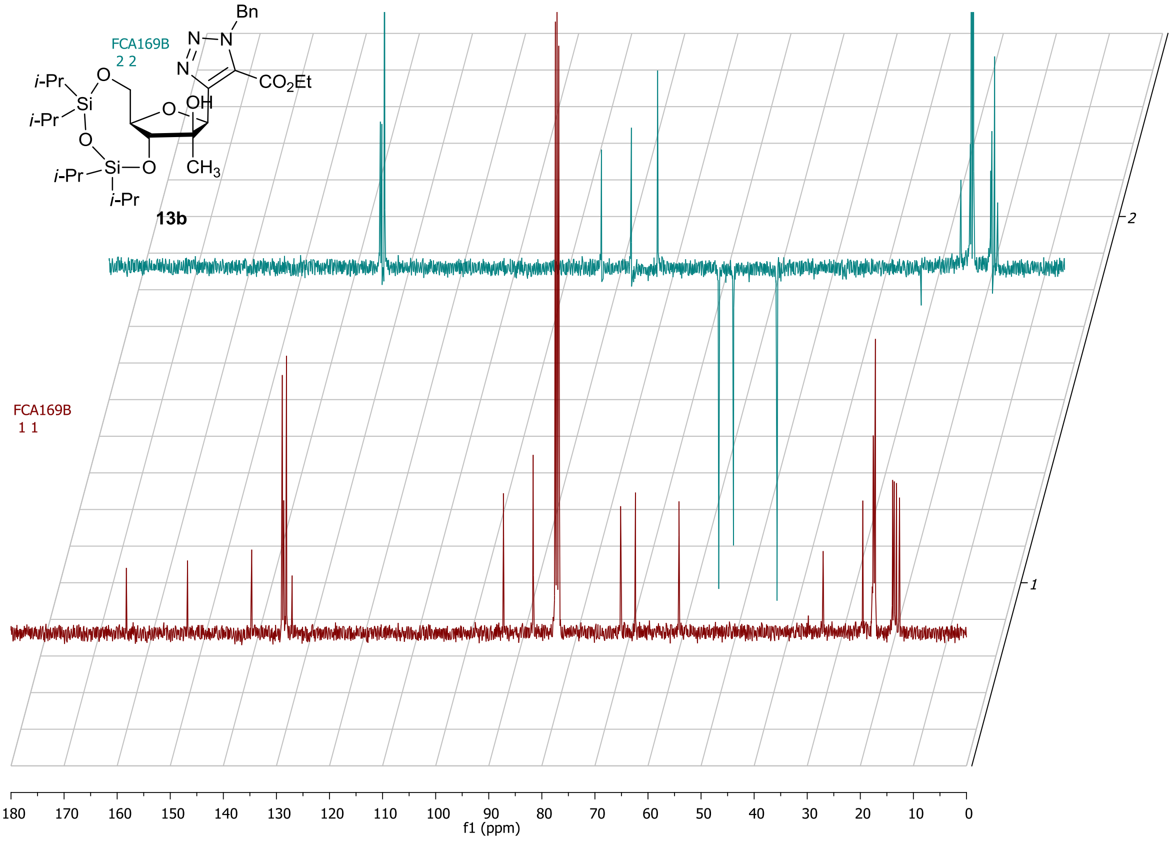

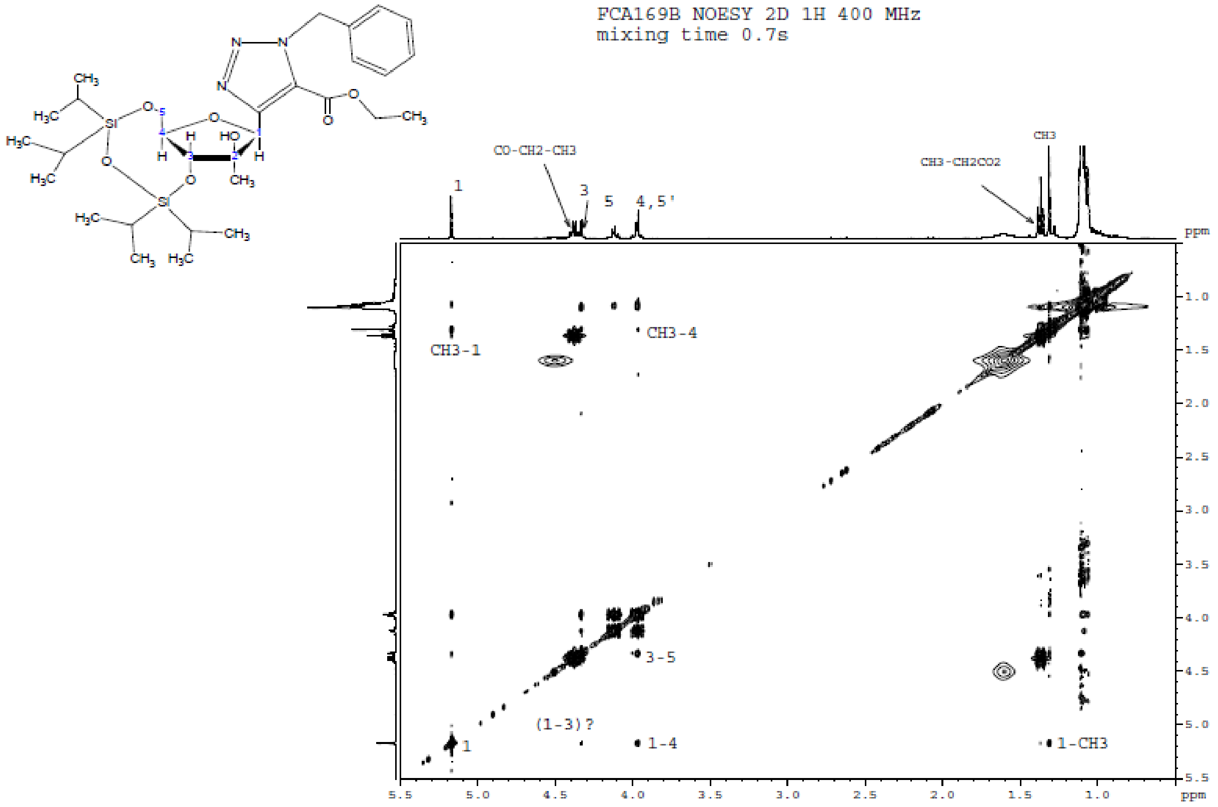

FCA169B NOESY 2D 1H 400 MHz
mixing time 0.7s
CO-CH2-CH3
CH3-CH2CO2
CH3
1
3
5
4,5'
CH3-1
CH3-4
3-5
(1-3)?
1-4
1-CH3
ppm

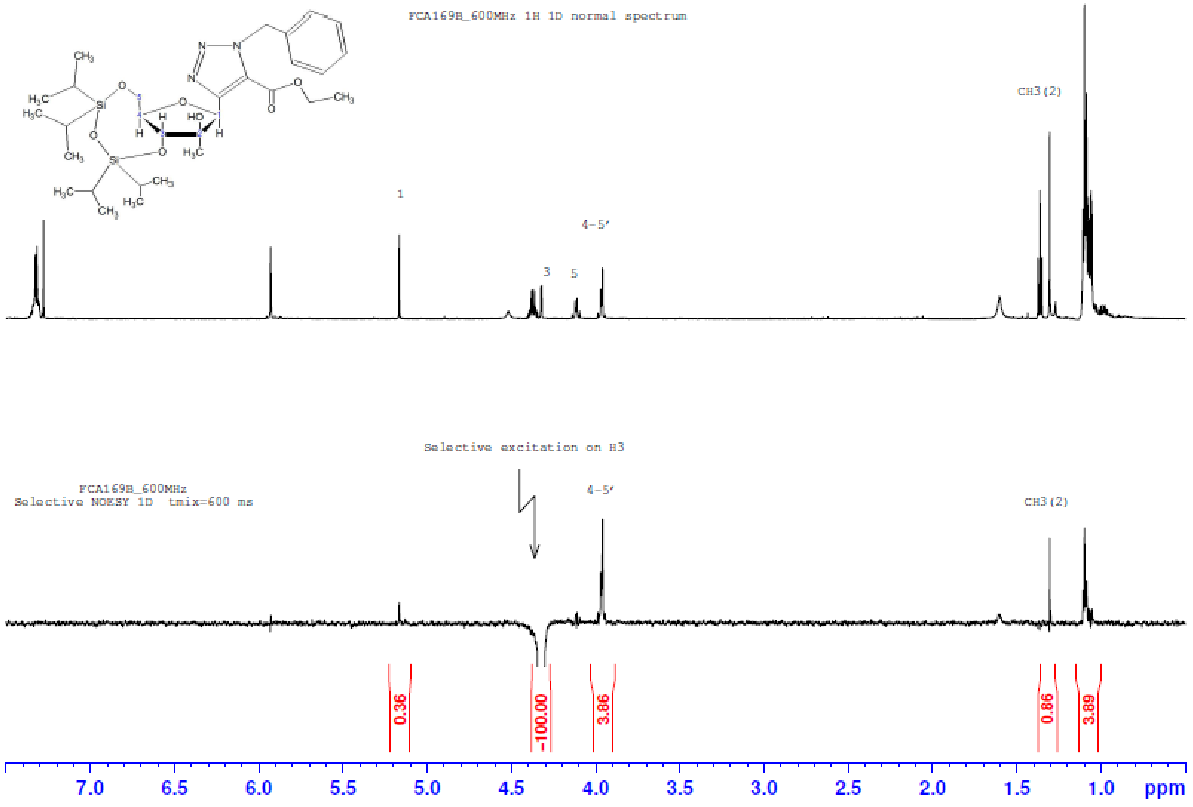

FCA169B_600MHz 1H 1D normal spectrum
1
3
5
4-5'
CH3(2)
FCA169B_600MHz
Selective NOESY 1D tmix=600 ms
Selective excitation on H3
4-5'
CH3(2)
0.36
-100.00
3.86
0.86
3.89
7.0
6.5
6.0
5.5
5.0
4.5
4.0
3.5
3.0
2.5
2.0
1.5
1.0
ppm

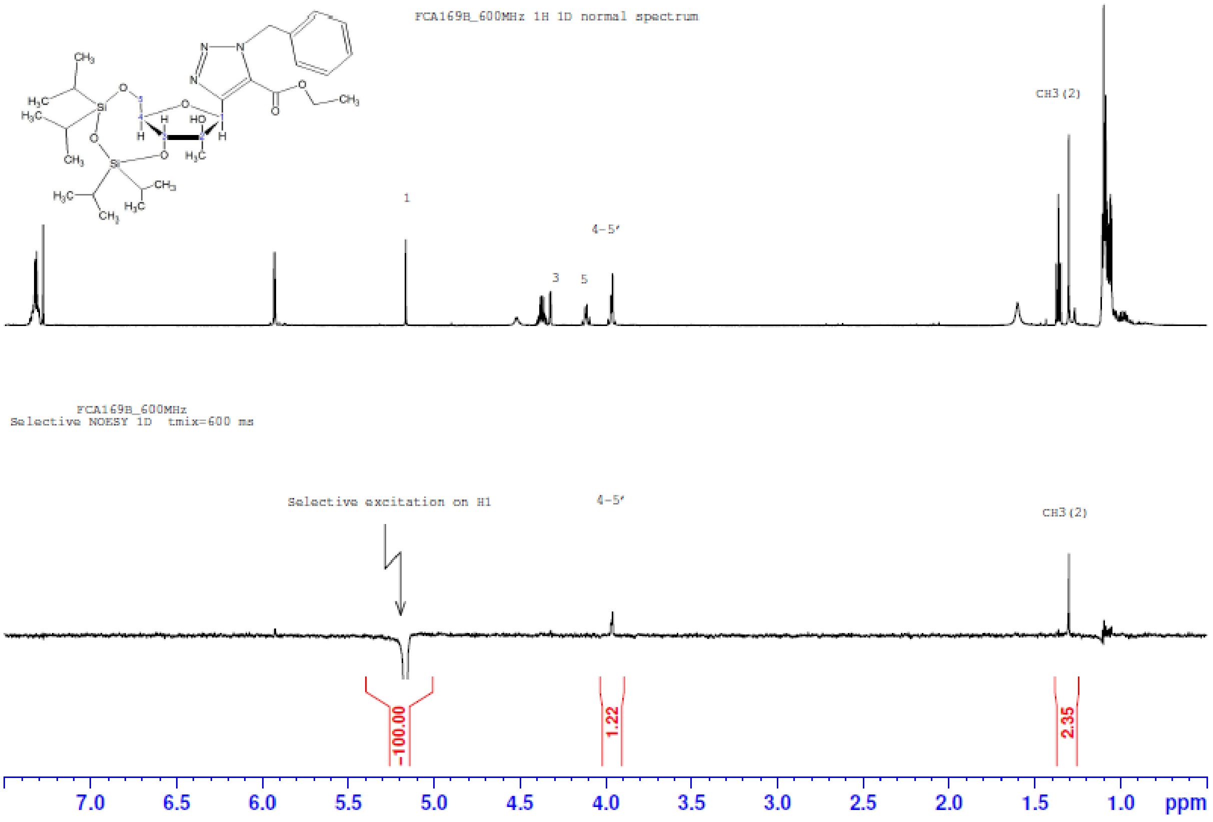
FCA169B_600MHz 1H 1D normal spectrum
1
3
5
4-5'
CH3(2)
FCA169B_600MHz
Selective NOESY 1D tmix=600 ms
Selective excitation on H1
4-5'
CH3(2)
-100.00
1.22
2.35
7.0
6.5
6.0
5.5
5.0
4.5
4.0
3.5
3.0
2.5
2.0
1.5
1.0
ppm

Ethyl 1-benzyl-4-(2’-deoxy-2’-fluoro-2’-methyl-3’,5’-*O*-(tetraisopropyldisiloxane-1,3-diyl)-β-D-ribofuranosyl)-1,2,3-triazole-5-carboxylate (14) and ethyl 1-benzyl-4-(2’-deoxy-2’-methylene-3’,5’-*O*-(tetraisopropyldisiloxane-1,3-diyl)-β-D-ribofuranosyl)-1,2,3-triazole-5-carboxylate (15)

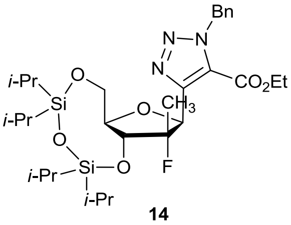

14

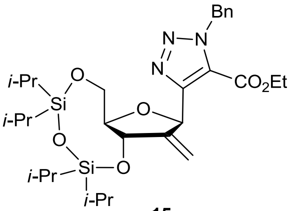

15

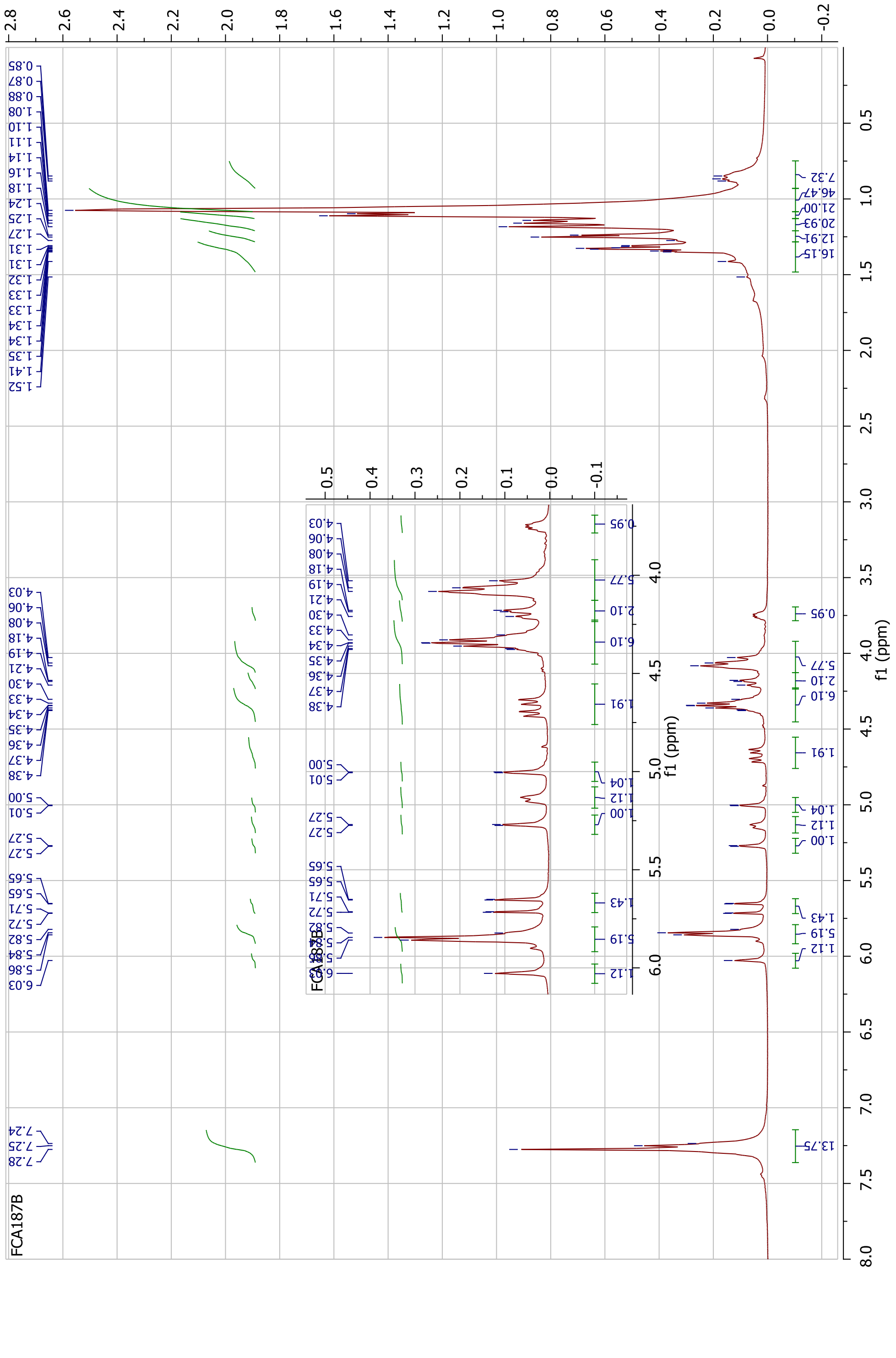

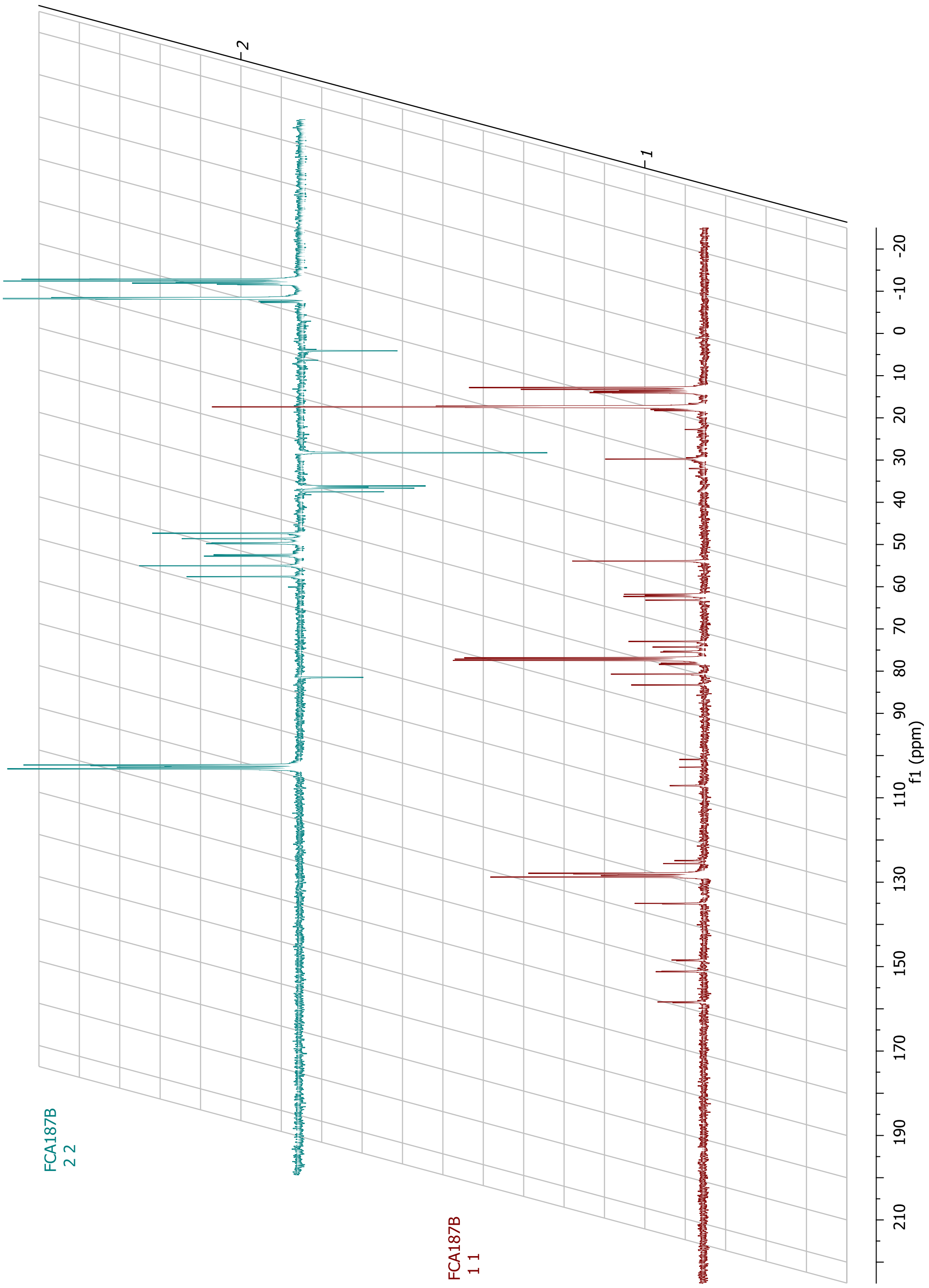
FCA187B
2 2
FCA187B
1 1
2
1
-20 -10 0 10 20 30 40 50 60 70 80 90 110 130 150 170 190 210
f1 (ppm)

Ethyl 1-benzyl-4-(2’-deoxy-2’-methyl-3’,5’-*O*-(tetraisopropyldisiloxane-1,3-diyl)-β-D-ribofur-1-enosyl)-1,2,3-triazole-5-carboxylate (16)

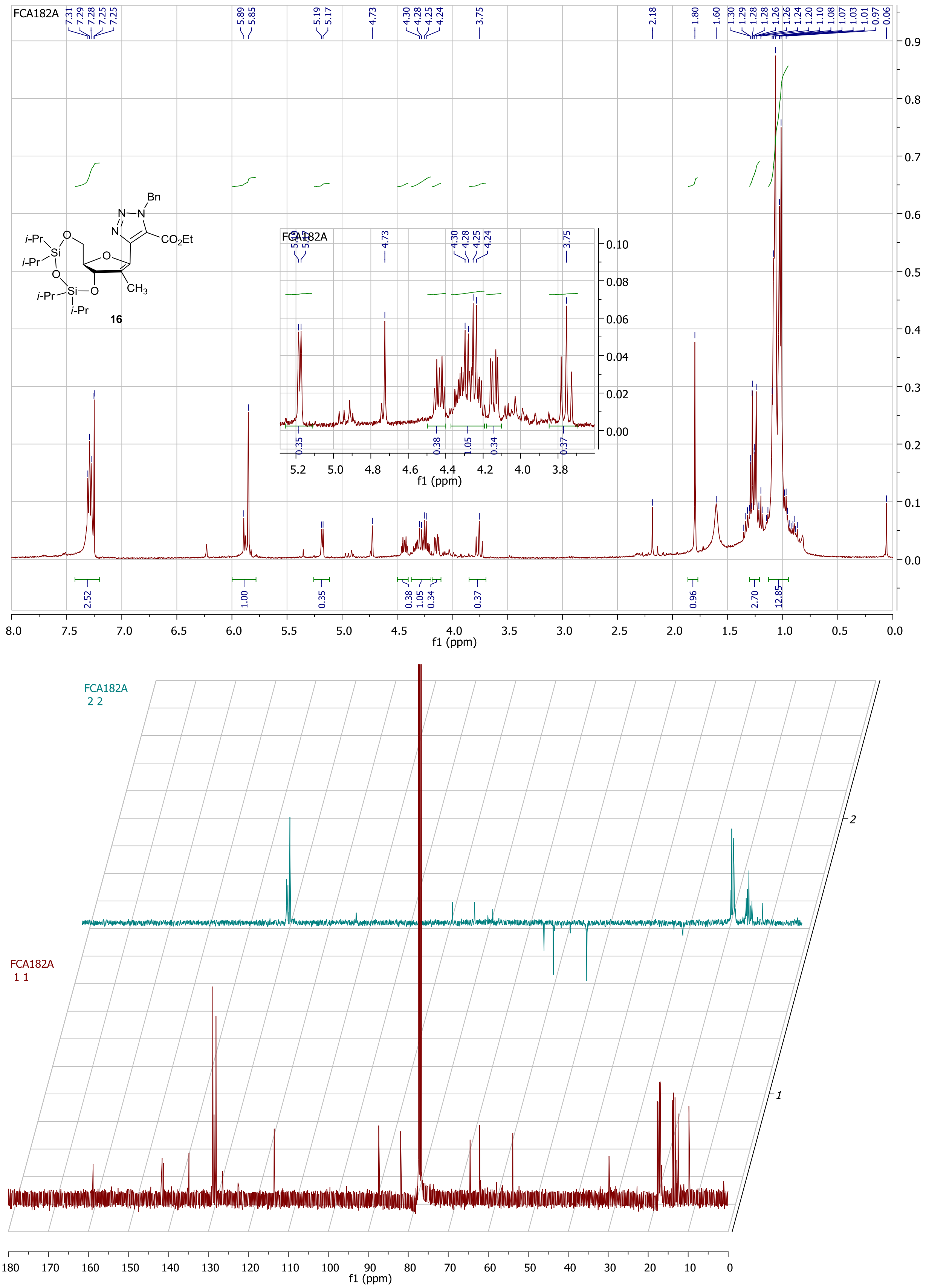

Ethyl 1-benzyl-4-(2’-deoxy-2’-fluoro-2’-methyl-β-D-ribofuranosyl)-1,2,3-triazole-5-carboxylate (17)

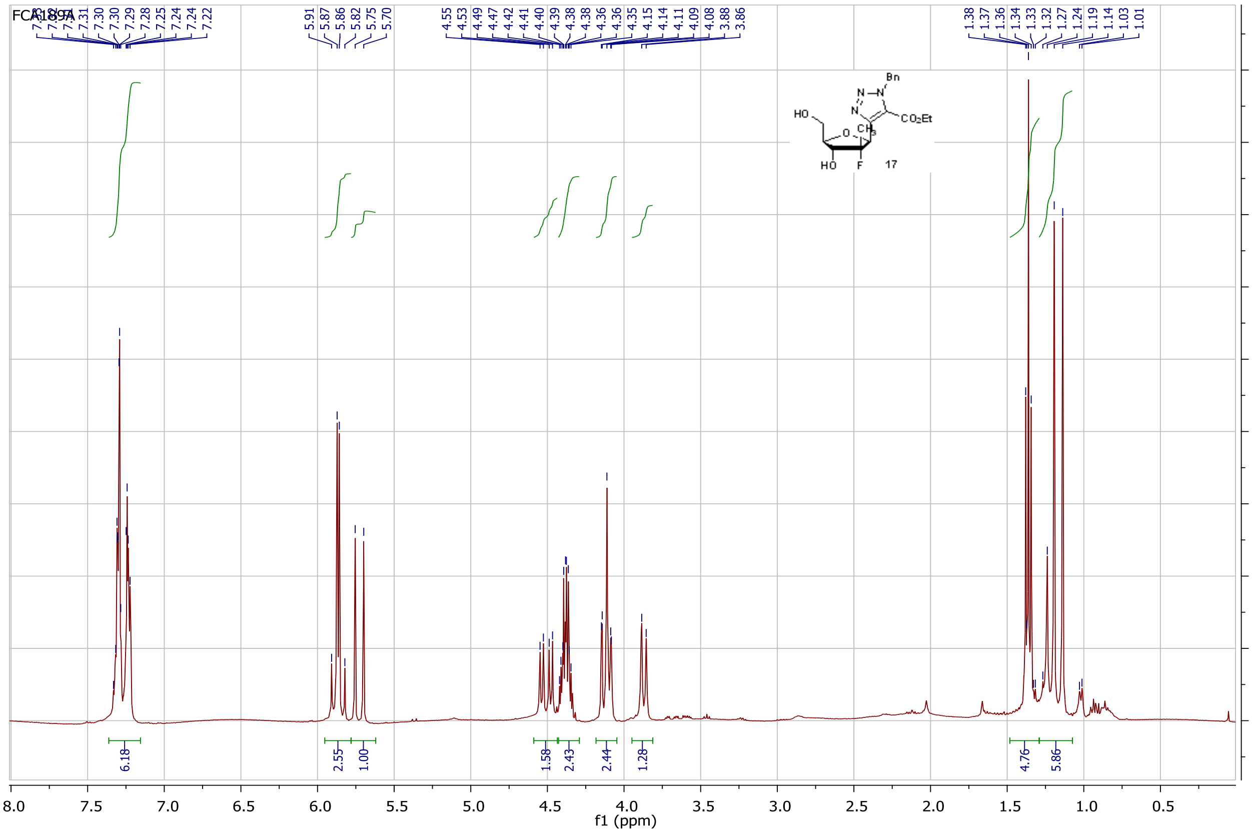

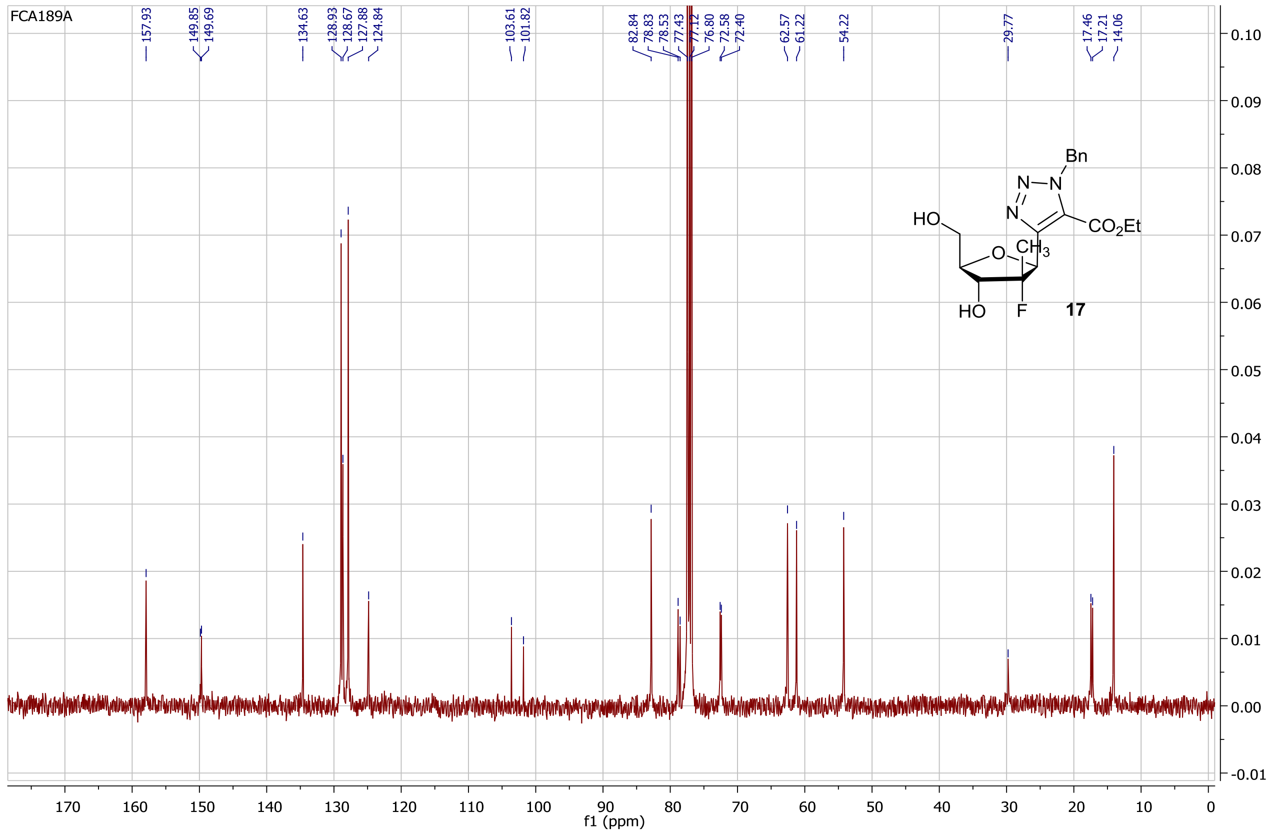

Ethyl 1-benzyl-4-(2’-deoxy-2’-methylene-β-D-ribofuranosyl)-1,2,3-triazole-5-carboxylate (18)

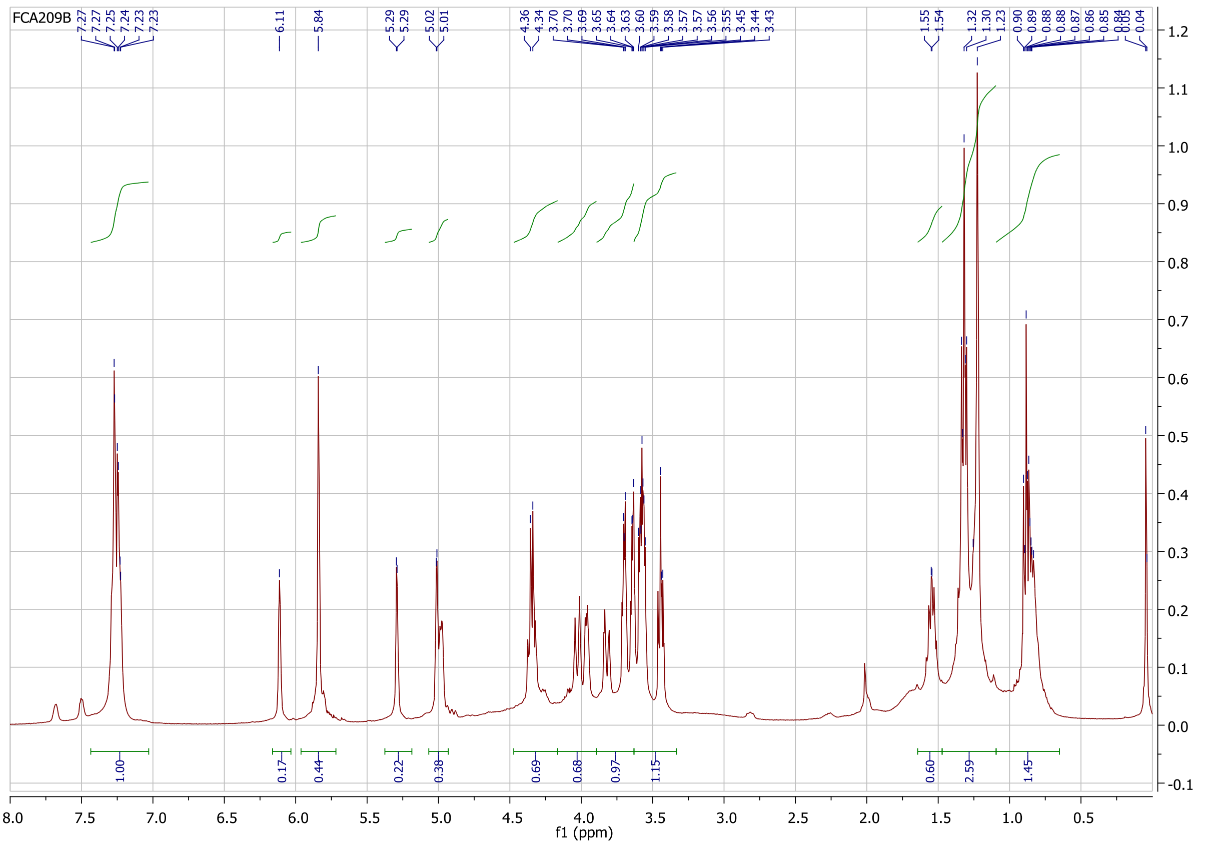

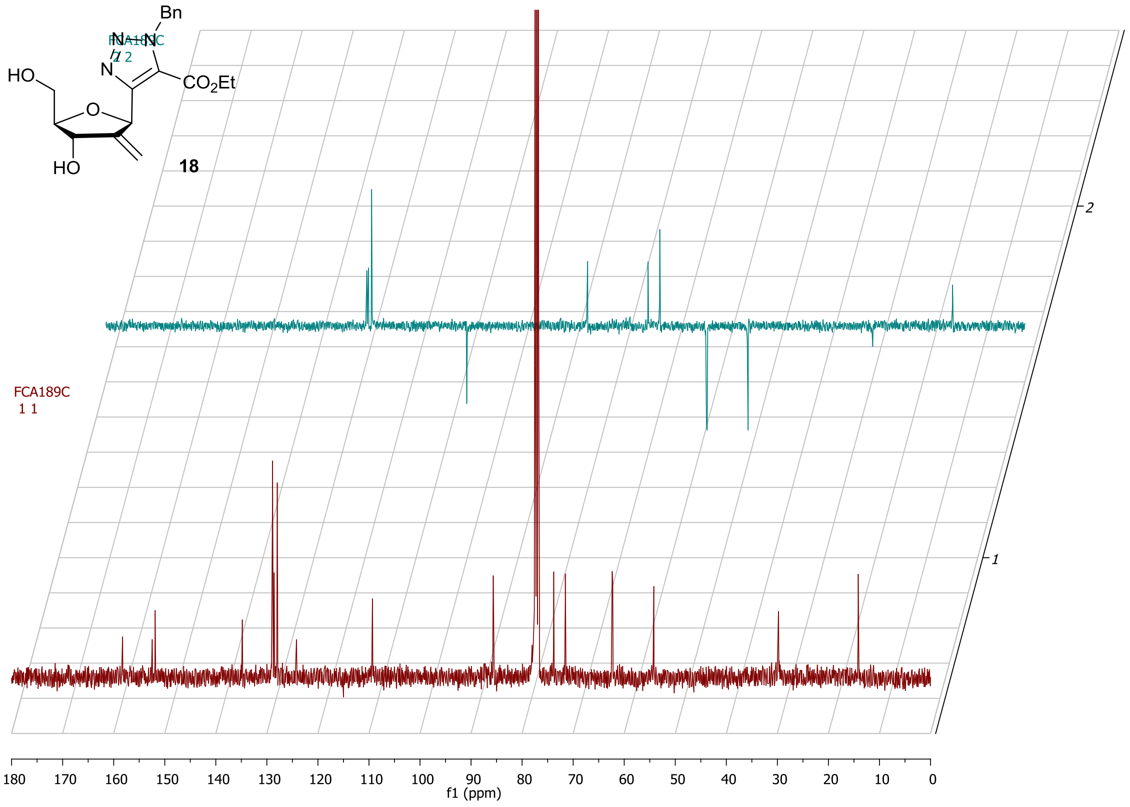

2’-Deoxy-2’-fluoro-2’-methyl-5-β-D-ribofuranosyl-1,2,3-triazole-4-carboxamide (3)

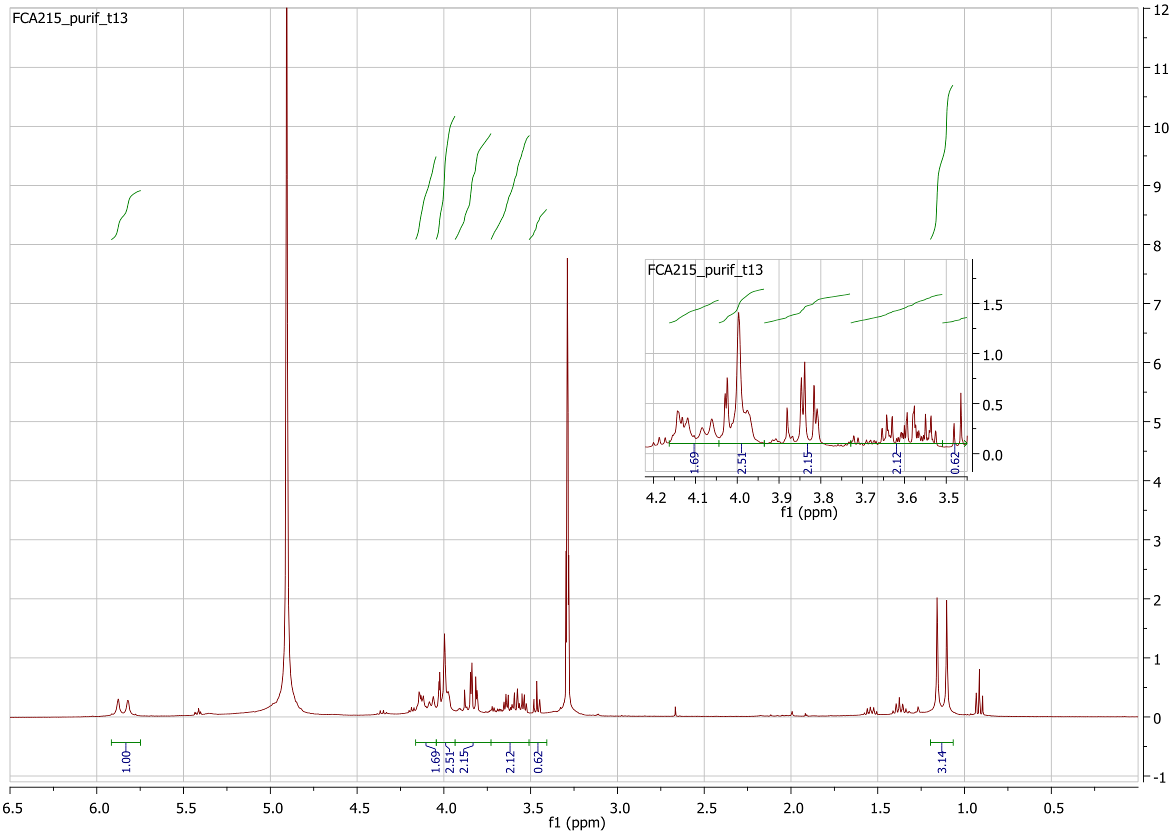

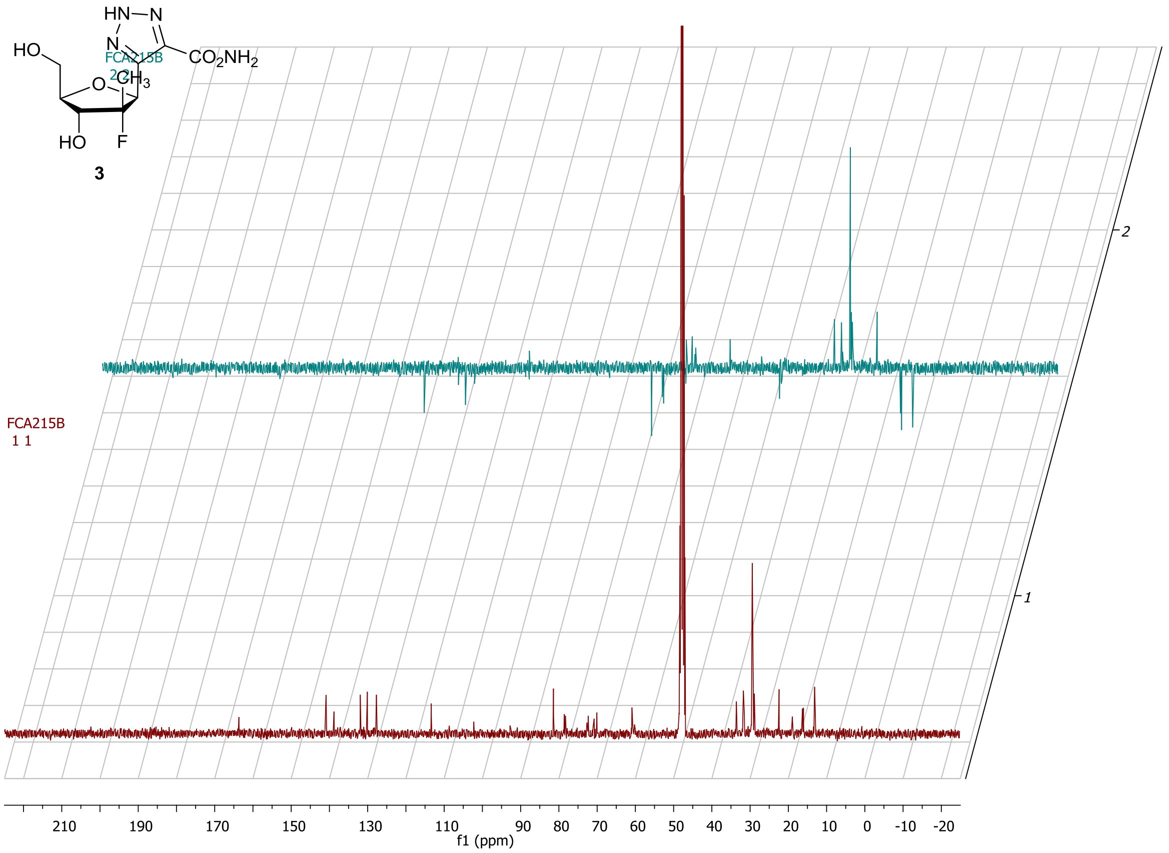

X-ray diffraction data was collected by using a Kappa X8 APPEX II Bruker diffractometer with graphite-monochromated MoKα radiation (λ = 0.71073 Å). Crystals were mounted on a CryoLoop (Hampton Research) with Paratone-N (Hampton Research) as cryoprotectant and then flash-frozen in a nitrogen-gas stream at 100 K. The temperature of the crystal was maintained at the selected temperature (100 K) by use of a 700 series Cryostream cooling device within an accuracy of ±1 K. The data were corrected for Lorentz polarization and absorption effects. The structures were solved by direct methods using SHELXS-97 [3] and refined against F^2 by full-matrix least-squares techniques using SHELXL-97 [4] with anisotropic displacement parameters for all non-hydrogen atoms. Hydrogen atoms were located on a difference Fourier map and introduced into the calculations as a riding model with isotropic thermal parameters. All calculations were performed by using the crystal structure crystallographic software package WINGX [5].

The crystal data collection and refinement parameters are given in Table S1.

CCDC 936439 contains the supplementary crystallographic data for this paper. These data can be obtained free of charge from the Cambridge Crystallographic Data Centre via www.ccdc.cam.ac.uk/data_ request/cif.
For **10b**, the C_6H_5 group is rotationally disordered with an occupancy ratio of 0.511(4) : 0.489(4).

Table S1: Crystallographic data and structure refinement details for compound **10b**.

**Compound**	**10b**
Formula	C_{16} H_{19} N_3 O_6
M_r	349.34
Crystal size, mm^3	0.14 x 0.03 x 0.01
Crystal system	orthorhombic
Space group	$P\ 2_1\,2_1\,2_1$
a, Å	4.9742(10)
b, Å	17.253(3)
c, Å	18.466(4
α, °	90
β, °	90
γ, °	90
Cell volume, $Å^3$	1584.7(5)
Z ; Z'	4 ; 1
T, K	100(1)
F_{000}	806
μ, mm^{-1}	0.113
θ range, °	1.62 – 30.61
Reflection collected	13 076
Reflections unique	4 217
R_{int}	0.1013
GOF	0.985
Refl. obs. (I>2σ(I))	2 072
Parameters	264
wR_2 (all data)	0.1530
R value (I>2σ(I))	0.0670
Largest diff. peak and hole (e-.$Å^{-3}$)	-0.337 ; 0.235

References

1. Cosson, F. PhD Thesis, University of Cergy-Pontoise, **2014**.
2. Aït Youcef, R., Dos Santos, M., Roussel, S., Baltaze, J.-P., Lubin-Germain, N., Uziel, J. *J. Org. Chem.* **2009**, *74*, 4318-4323.
3. Sheldrick, G. M. SHELXS-97, Program for Crystal Structure Solution, University of Göttingen, Göttingen, Germany, **1997**.
4. Sheldrick, G. M. SHELXL-97, Program for the refinement of crystal structures from diffraction data, University of Göttingen, Göttingen, Germany, **1997**.
5. Farrugia, L. J. *J. Appl. Cryst*. **1999**, *32*, 837.
